# Supplementary material for: Halting predicted vertebrate declines requires tackling multiple drivers of biodiversity loss
Source: Sci Adv. 2026 Feb 11;12(7):eadx7973. doi: 10.1126/sciadv.adx7973 (PMC12893280; doi:10.1126/sciadv.adx7973)
Supplement: Supplementary file 1 — Figs. S1 to S17 Tables S1 to S6 [file sciadv.adx7973_sm.pdf]

Science Advances
AAAS

Supplementary Materials for

Halting predicted vertebrate declines requires tackling multiple drivers of biodiversity loss

Pol Capdevila *et al.*

Corresponding author: Pol Capdevila, pcapdevila@ub.edu; Duncan O'Brien, duncan.obrien@bristol.ac.uk

Sci. Adv. **12**, eadx7973 (2026)
DOI: 10.1126/sciadv.adx7973

This PDF file includes:

Figs. S1 to S17
Tables S1 to S6

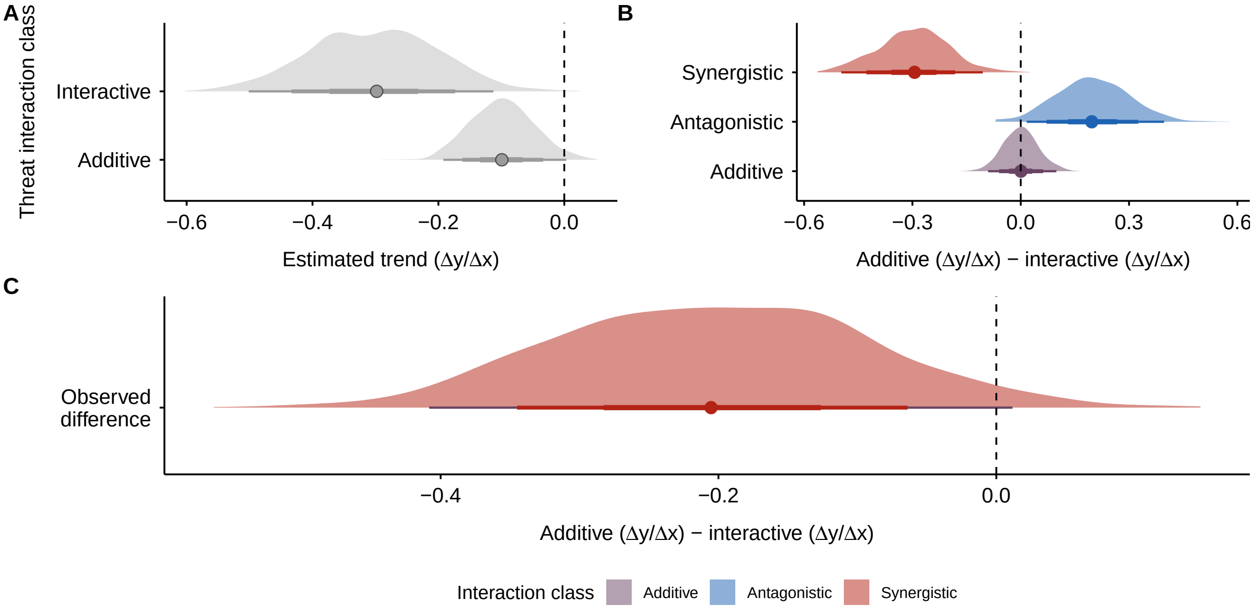

Figure S1: Demonstration of the threat classification protocol. (A) Trends are first predicted for each threat in additive or interactive combinations. (B) When the difference between predicted interactive trends and additive trends is calculated, there are three possible classes: additive (no difference in predictions made by additive and interacting threats), antagonistic (interacting threat predictions are less negative than additive predictions), or synergistic (interacting threat predictions are more negative than additive predictions). Classes are defined using the 80% credible interval. We consider that a synergy 'worsens' trends - i.e. becomes more negative. (C) The actual difference between predictions made in panel A.

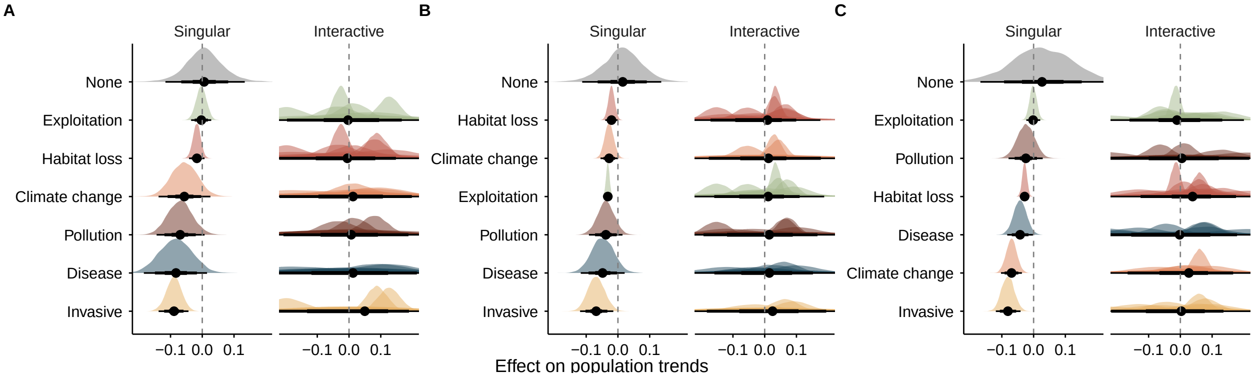

Figure S2: Effects of threats on vertebrate population trends across realms. 95% credible intervals of the effects of single and interacting threats upon (A) freshwater, (B) marine, and (C) terrestrial time series trends. The dashed vertical line shows zero influence – i.e., no effect of the factors – while the None parameter is the trend in the absence of threats. The remaining parameters represent modifications of this None trend. The credible intervals are based on 1,000 samples from the posterior distribution of the model coefficients.

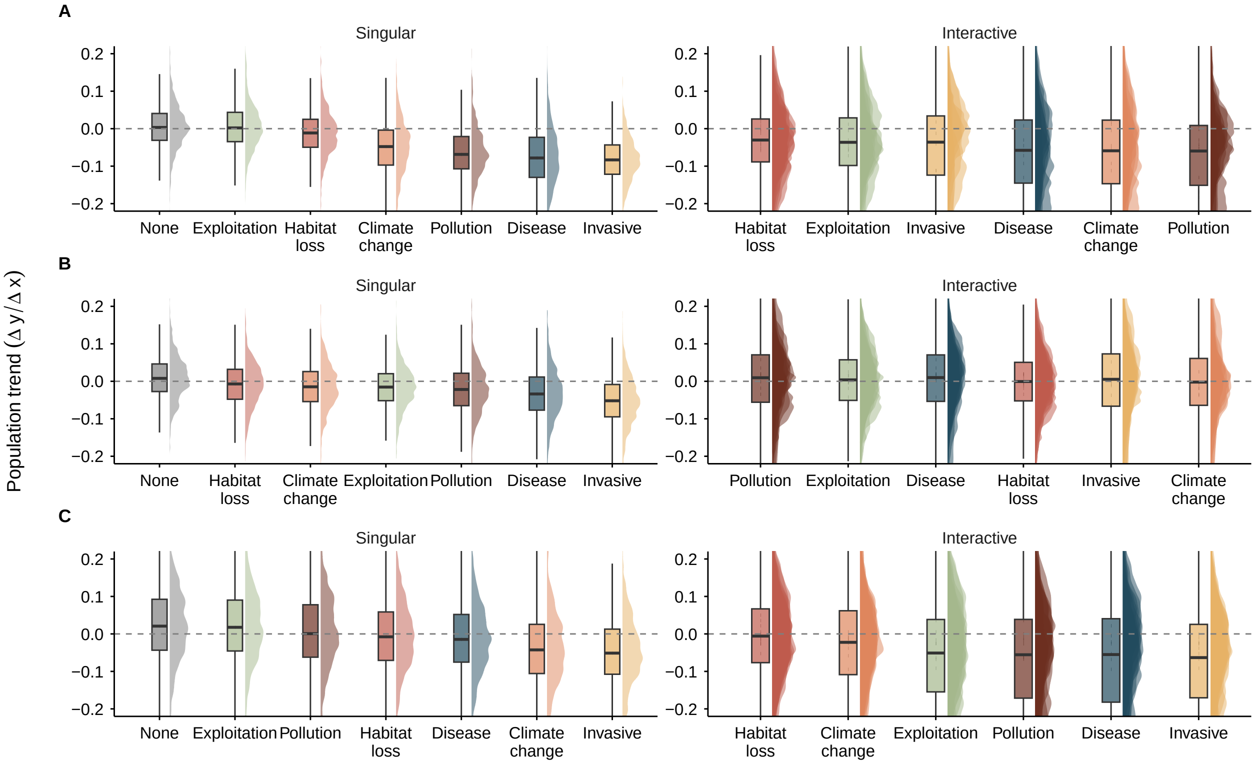

Figure S3: Ultimate effect of threats on vertebrate population trends across realms. 95% credible intervals of the ultimate estimated trend in the presence of single and interacting threats for (A) freshwater, (B) marine, and (C) terrestrial time series. The dashed horizontal line shows the zero-slope – i.e., no effect of the factors. The credible intervals are based on 1,000 samples from the posterior distribution of the model predictions where threats are included/excluded from the model matrix.

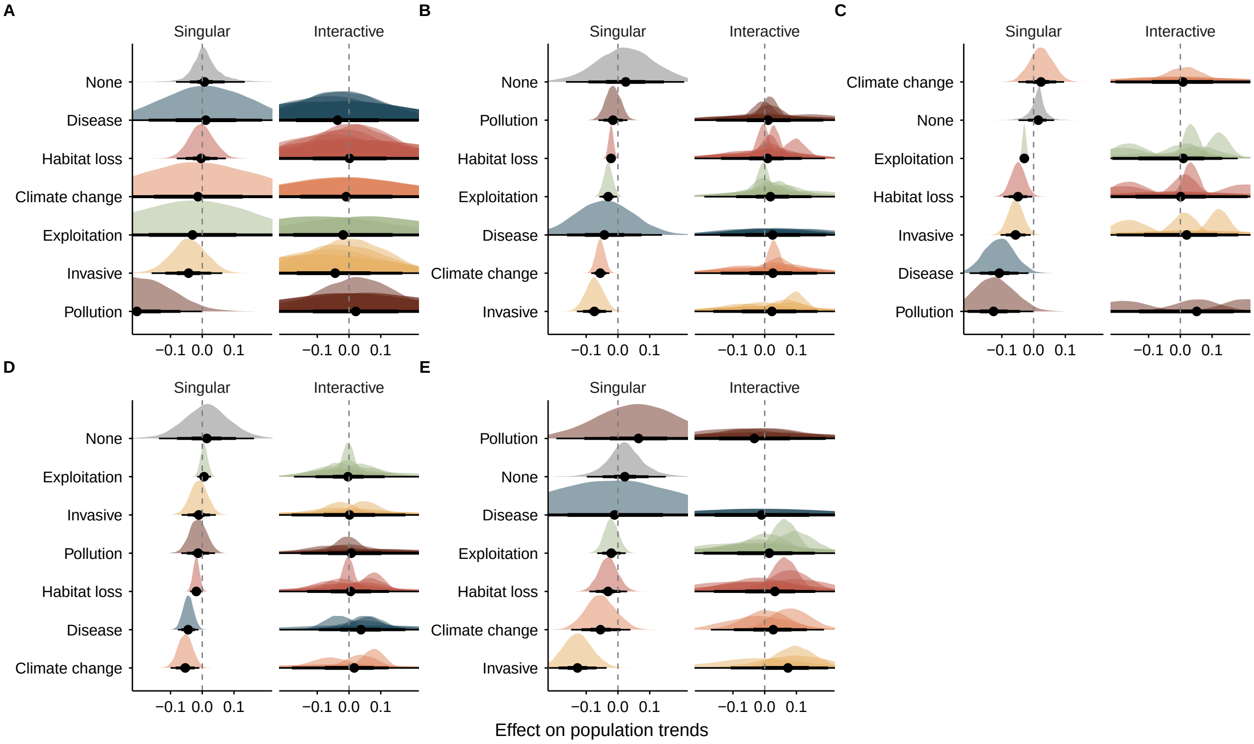

Figure S4: Effects of threats on vertebrate population trends across taxa. 95 % credible intervals of the effects of single and interacting threats upon (A) amphibian, (B) bird, (C) fish, (D) mammalian, and (E) reptilian time series trends. The dashed vertical line shows zero influence – i.e., no effect of the factors – while the None parameter is the trend in the absence of threats. The remaining parameters represent modifications of this None trend. The credible intervals are based on 1,000 samples from the posterior distribution of the model coefficients.

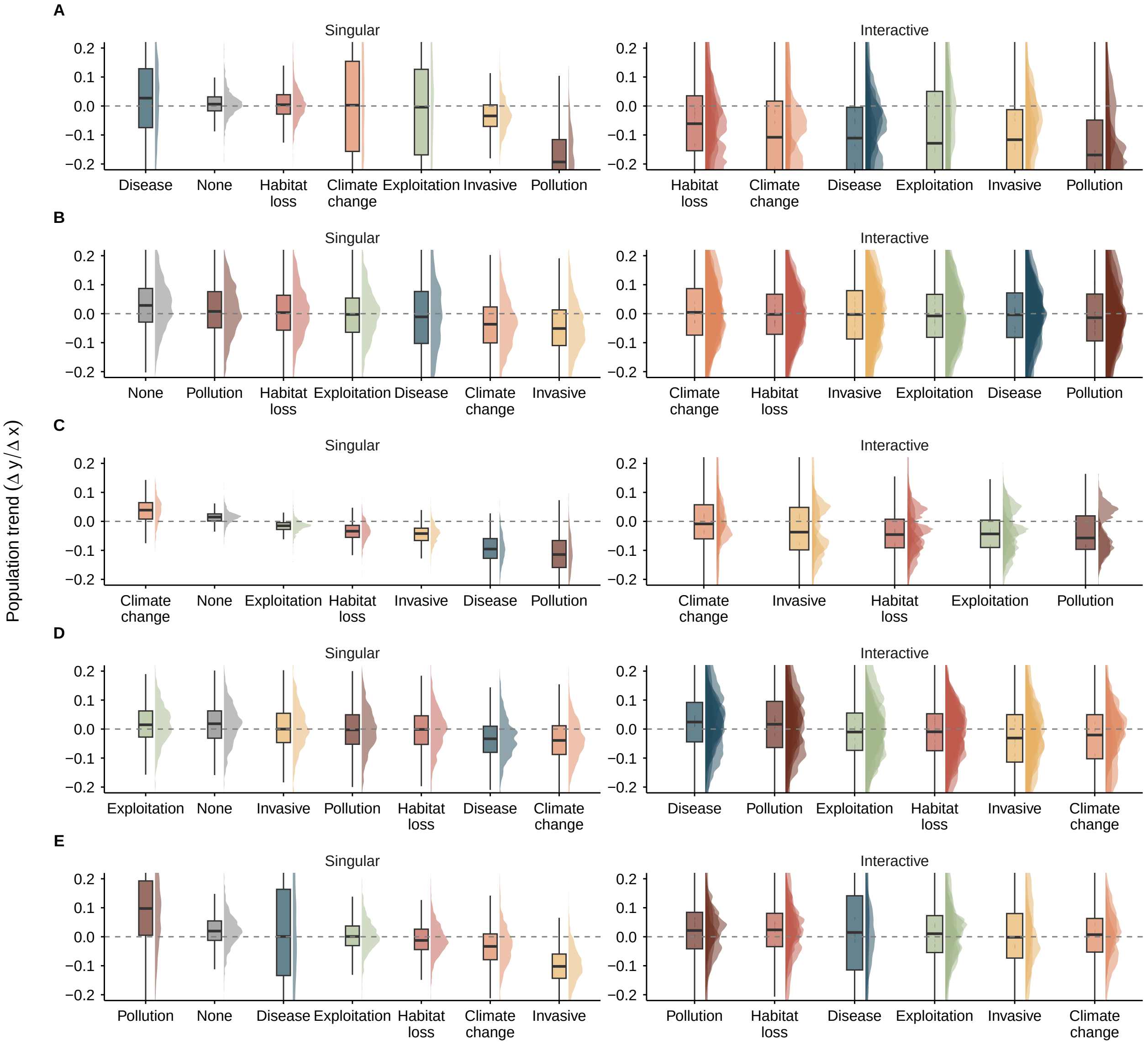

Figure S5: Ultimate effect of threats on vertebrate population trends across taxa. 95 % credible intervals of ultimate estimated trends in the presence of single and interacting threats for (A) amphibian, (B) bird, (C) fish, (D) mammalian, and (E) reptilian. The dashed horizontal line shows the zero-slope – i.e., no effect of the factors. The credible intervals are based on 1,000 samples from the posterior distribution of the model predictions where threats are included/excluded from the model matrix.

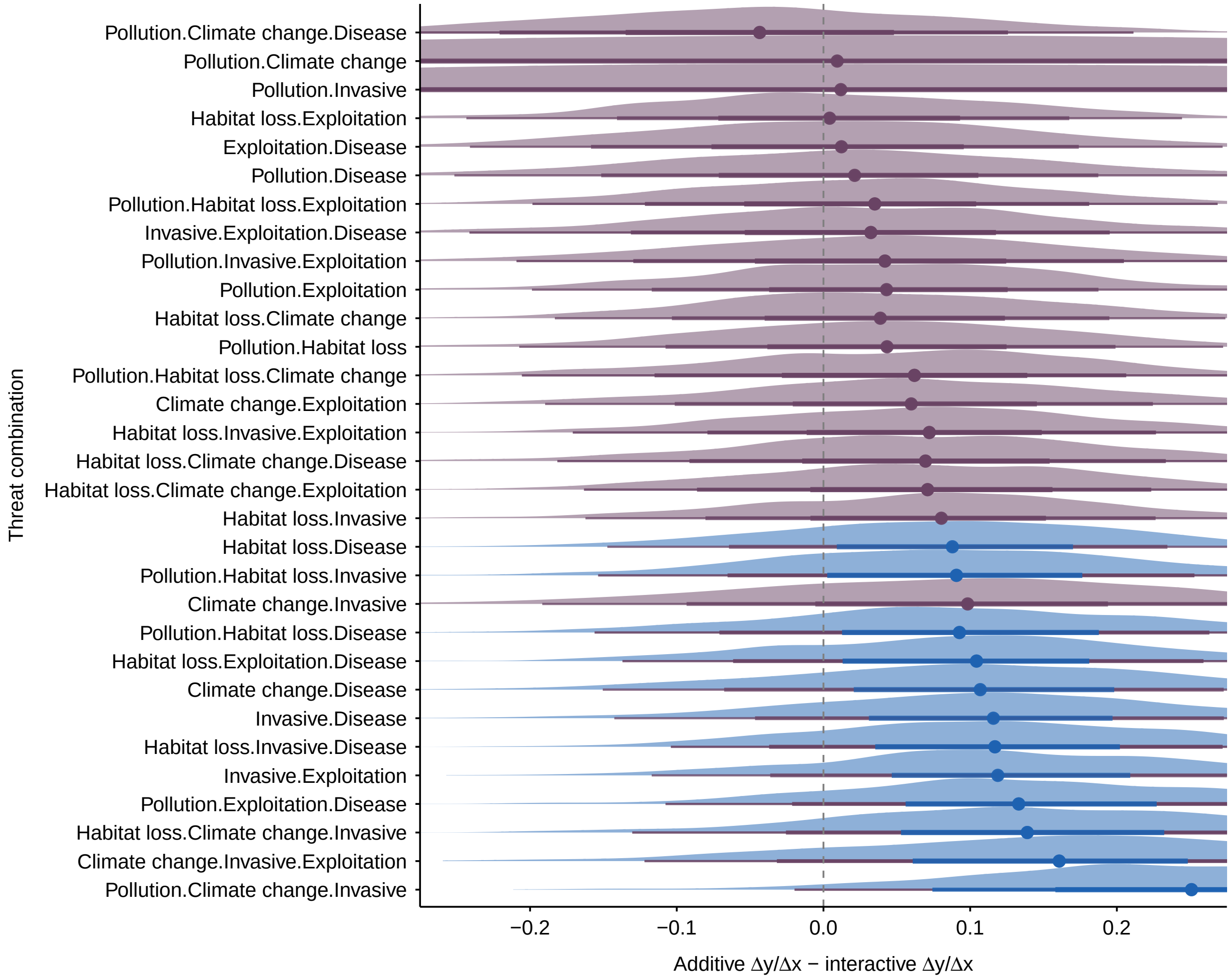

Figure S6: Definition of interacting threat classes using 1000 posterior draws estimated from additive vs interacting model matrices. The difference between additive and interacting trend posteriors is calculated with zero differences assumed to be additive. If the 80 % credible interval does not intersect 0, and is negative, then that threat combination is assumed to be synergistic, whereas if positive, the combination is classified as antagonistic.

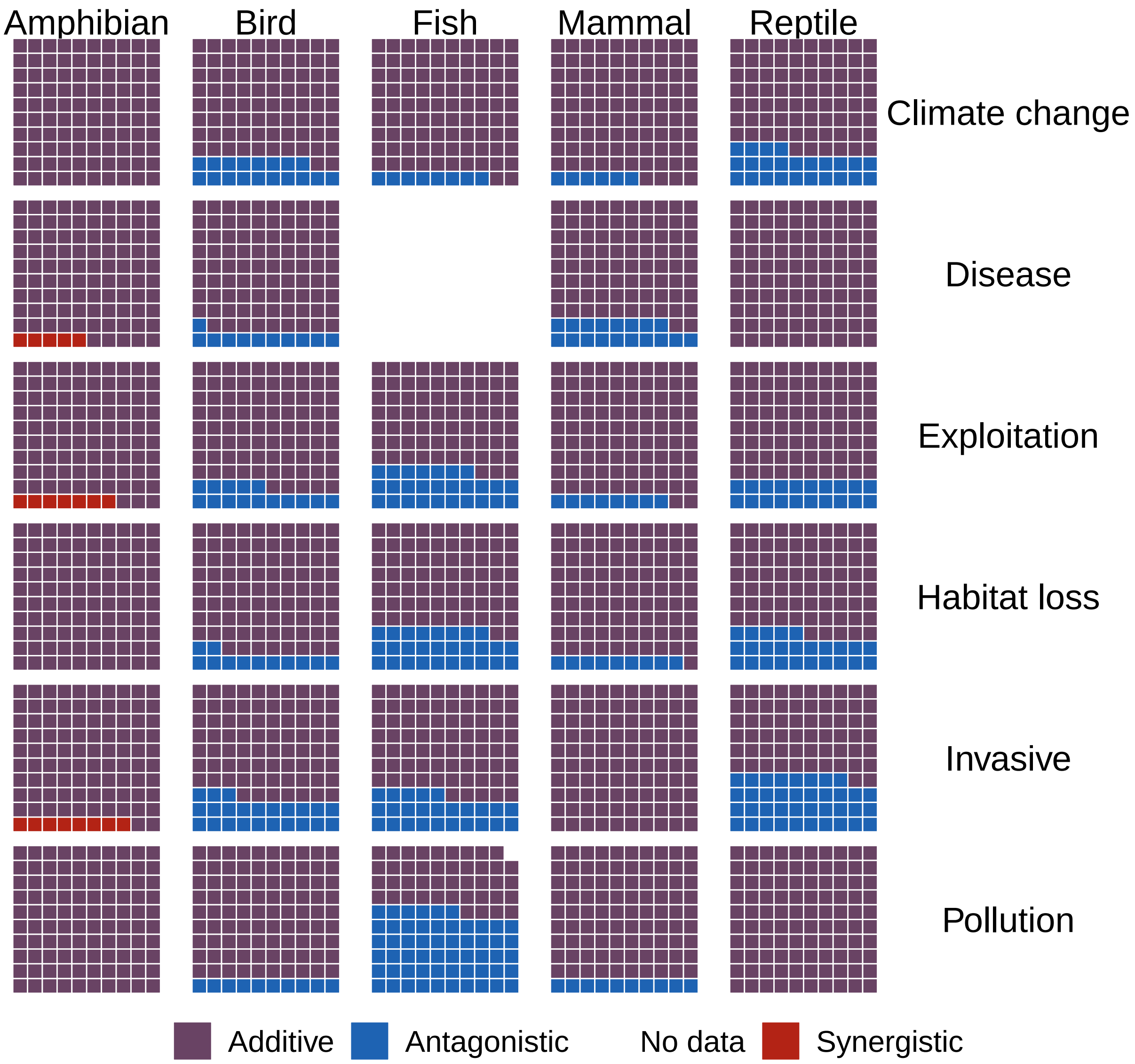

Figure S7: Spread of interactive effects across threats and taxa.

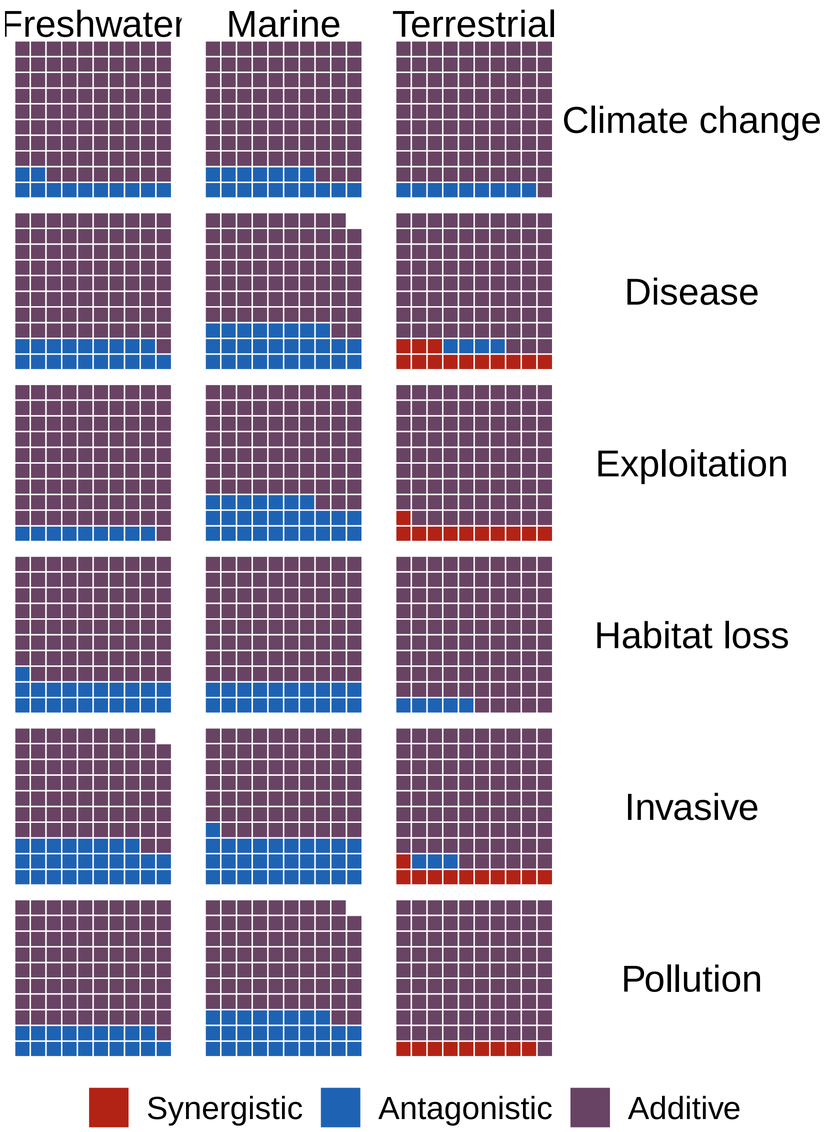

Figure S8: Spread of interactive effects across threats and systems.

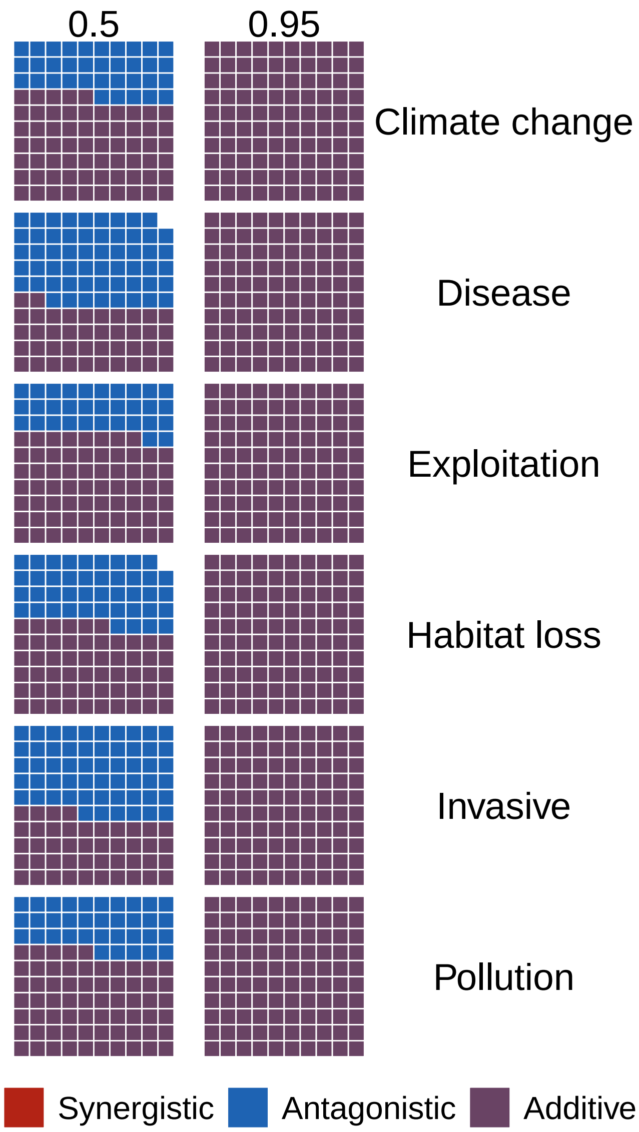

Figure S9: Demonstration of how changing the credible interval from 50 to 95% alters the classification of each threat combination.

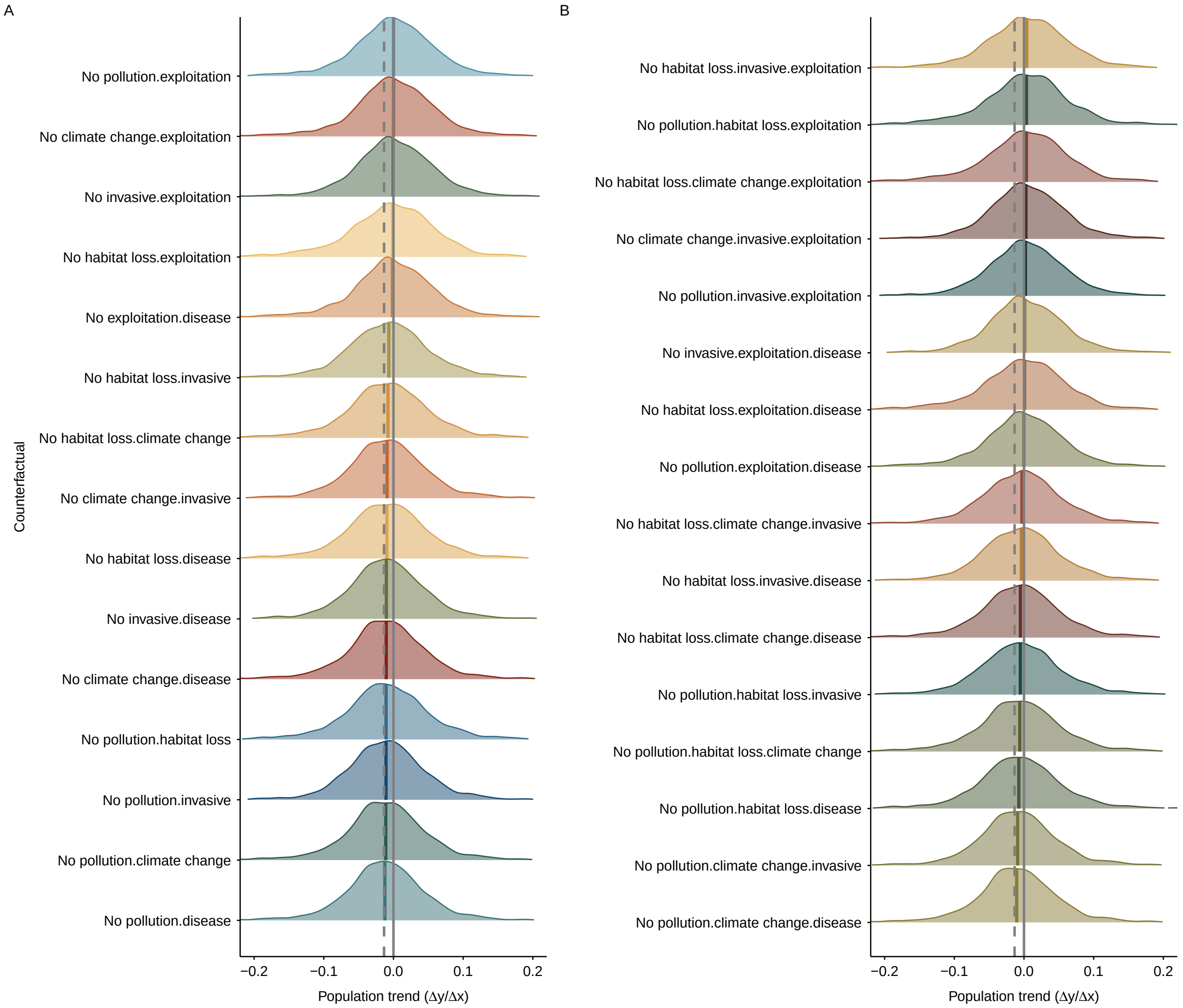

Figure S10: Counterfactual predictions for multiple threats. The counterfactuals represent the changes in the population trends of the 1,740 vertebrate time-series affected by threats, had there been different combinations of multiple threats. (A) Counterfactuals representing the global vertebrate population growth where different combinations of two threats were removed. (B) Counterfactuals representing the global vertebrate population growth where three threats were removed. The black line represents when the population trend is 0. The dotted line is the median trend of the threatened populations without any intervention.

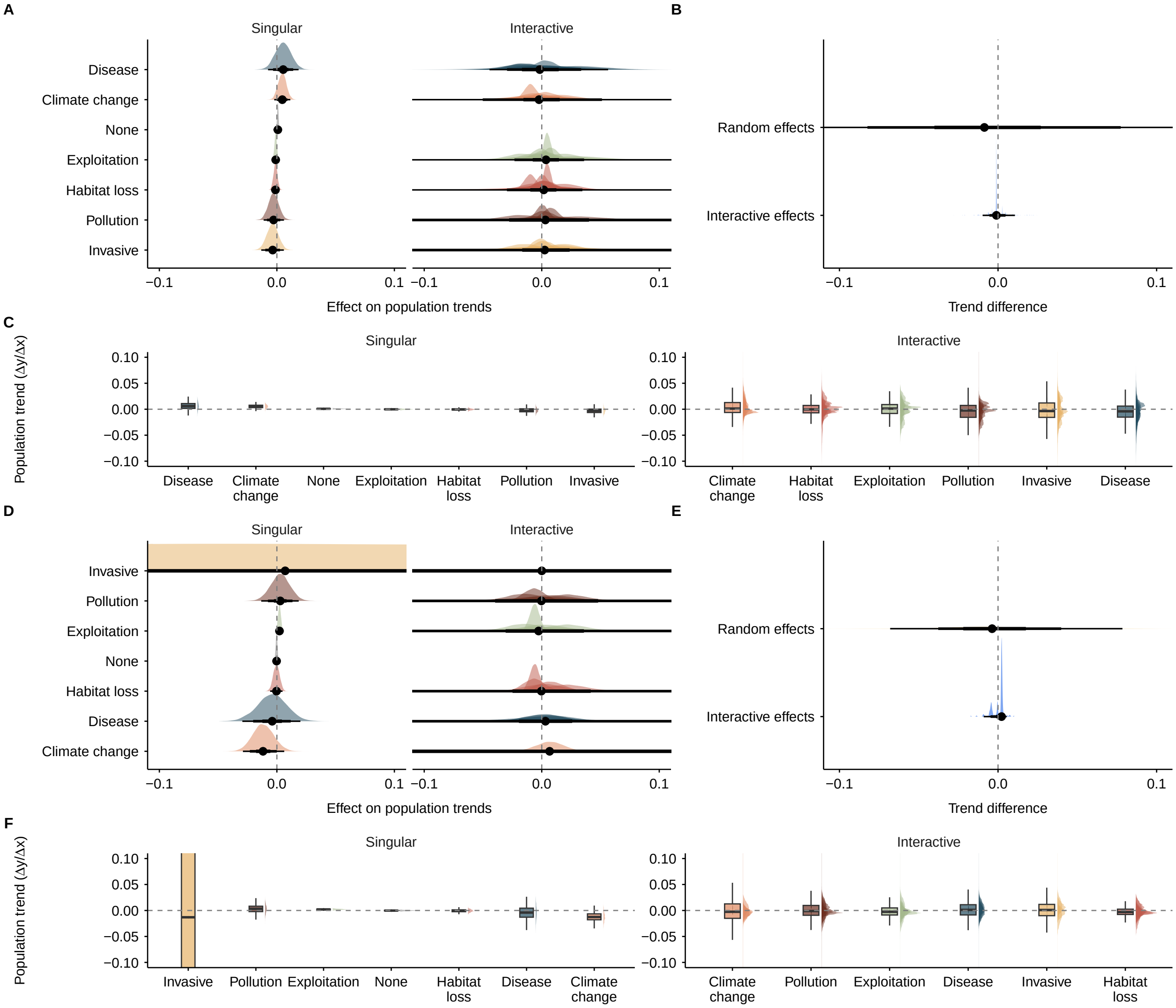

Figure S11: Influence of time series length on the relationship between threats and Living Planet Database trends. (A-C) Model estimates for time series containing 10 years of data. (D-F) Model estimates for time series containing 20 years of data.

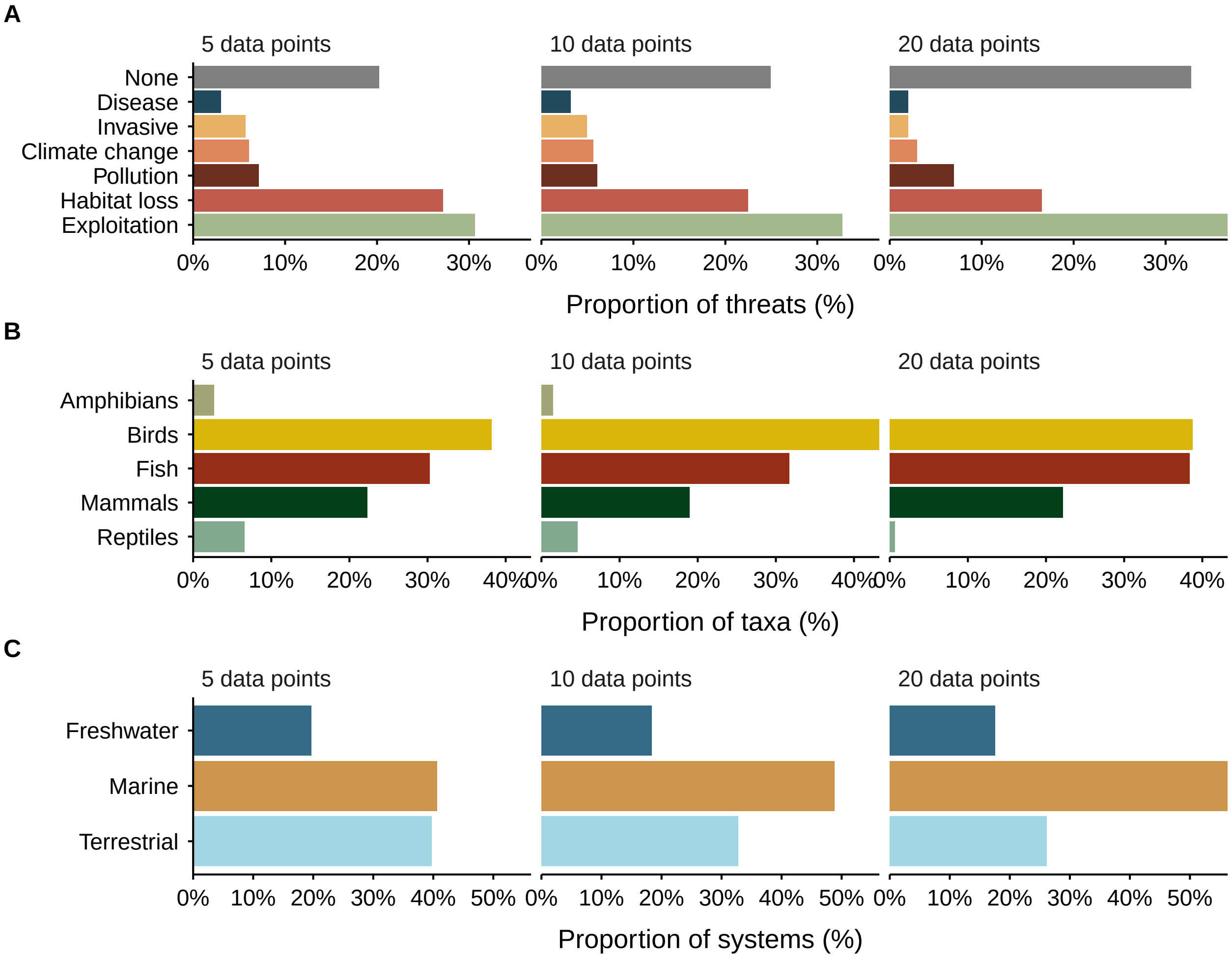

Figure S12: Frequency of threats, systems and taxa in the Living Planet Database once records have been filtered to time series containing 5, 10 and 20 years of data.

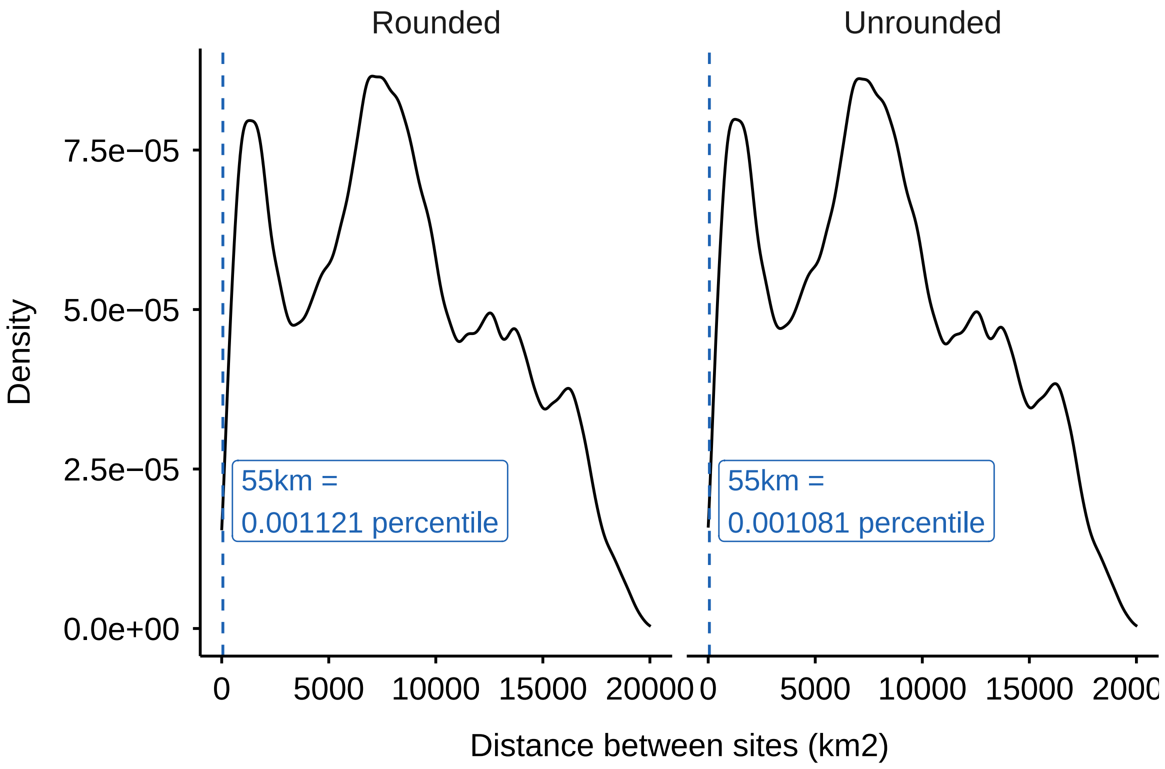

Figure S13: Influence of rounding latitude to the nearest degree upon the estimated distance between Living Planet Database sites.

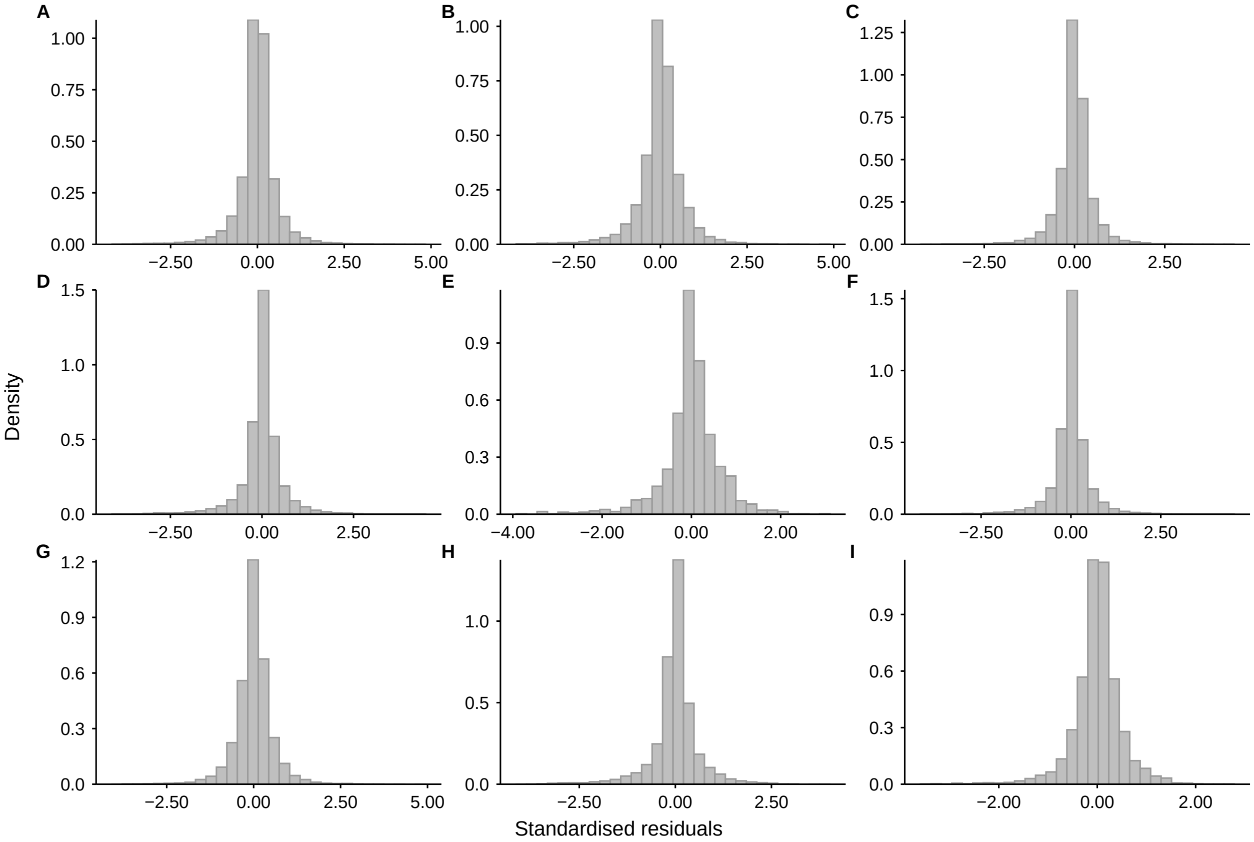

Figure S14: Distribution of the standardised residuals for the multilevel Bayesian models. Residuals were examined for the (A) global model, (B-D) system specific models (freshwater, marine, terrestrial), and (E-I) amphibians, birds, fishes, mammals, and reptiles respectively.

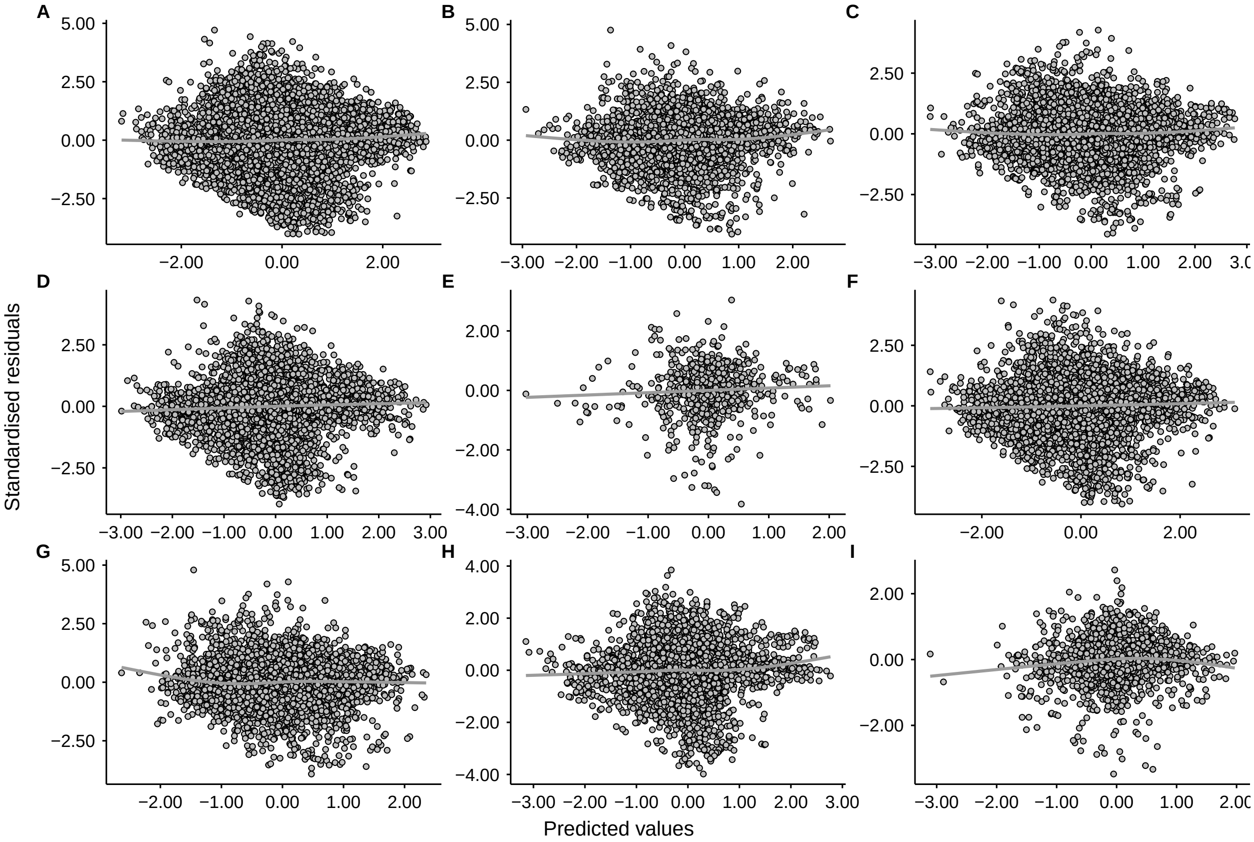

Figure S15: The standardised residuals vs the predicted values for the multilevel Bayesian models. The standardised residuals vs the predicted values have similar variances for all models, suggesting that the equal variance assumption is met. Residuals were examined for the (A) global model, (B-D) system specific models (freshwater, marine, terrestrial), and (E-I) amphibians, birds, fishes, mammals, and reptiles respectively.

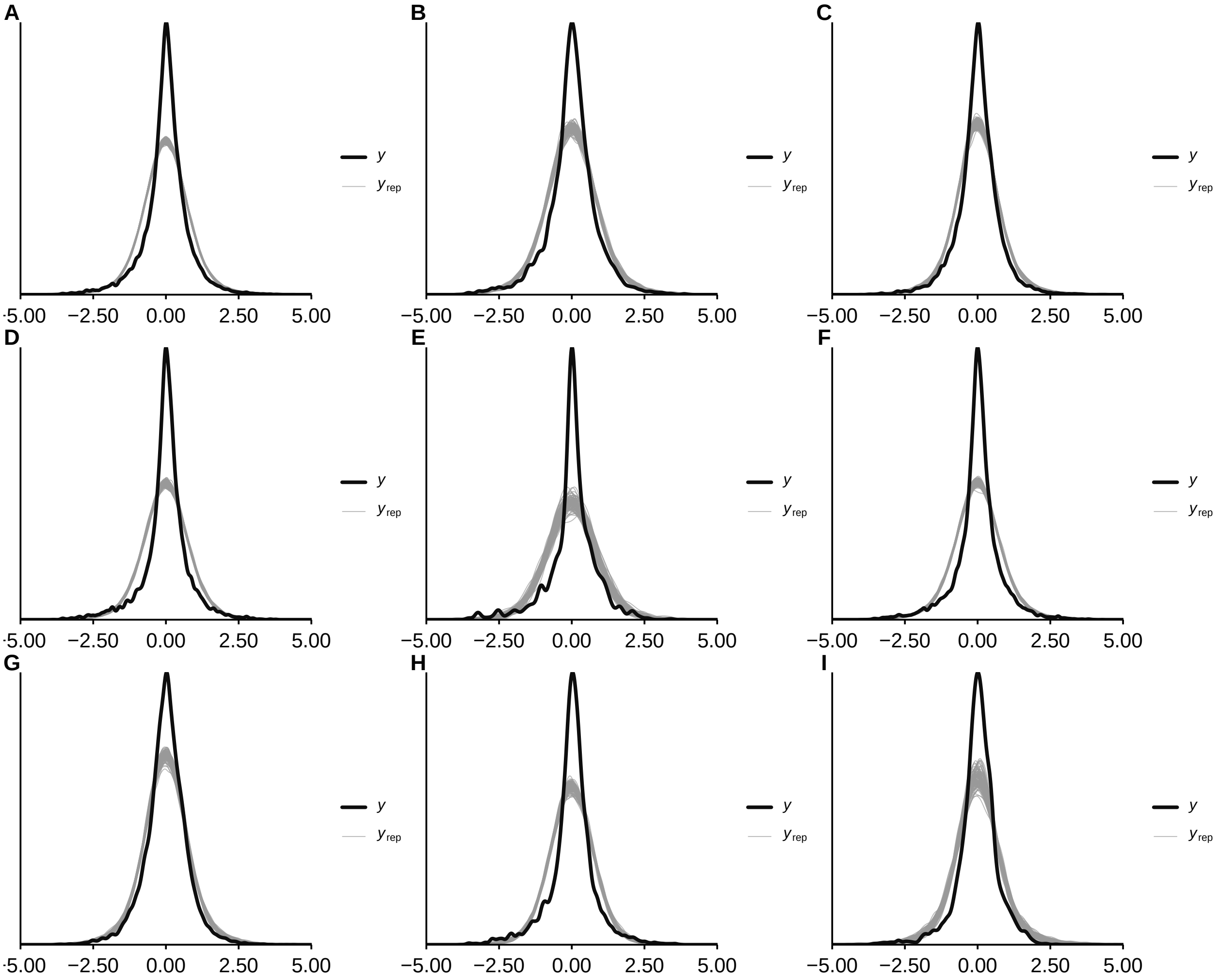

Figure S16: The posterior predictive checks for the multilevel Bayesian models. The posterior predictive checks do not show strong discrepancies between our data (dark lines, y) from the predictions from the model (light grey lines, yrep) for any of the models. However, the model shows a slight underestimation of the true zero values. The posterior predictive checks were examined for the (A) global model, (B-D) system specific models (freshwater, marine, terrestrial), and (E-I) amphibians, birds, fishes, mammals, and reptiles respectively.

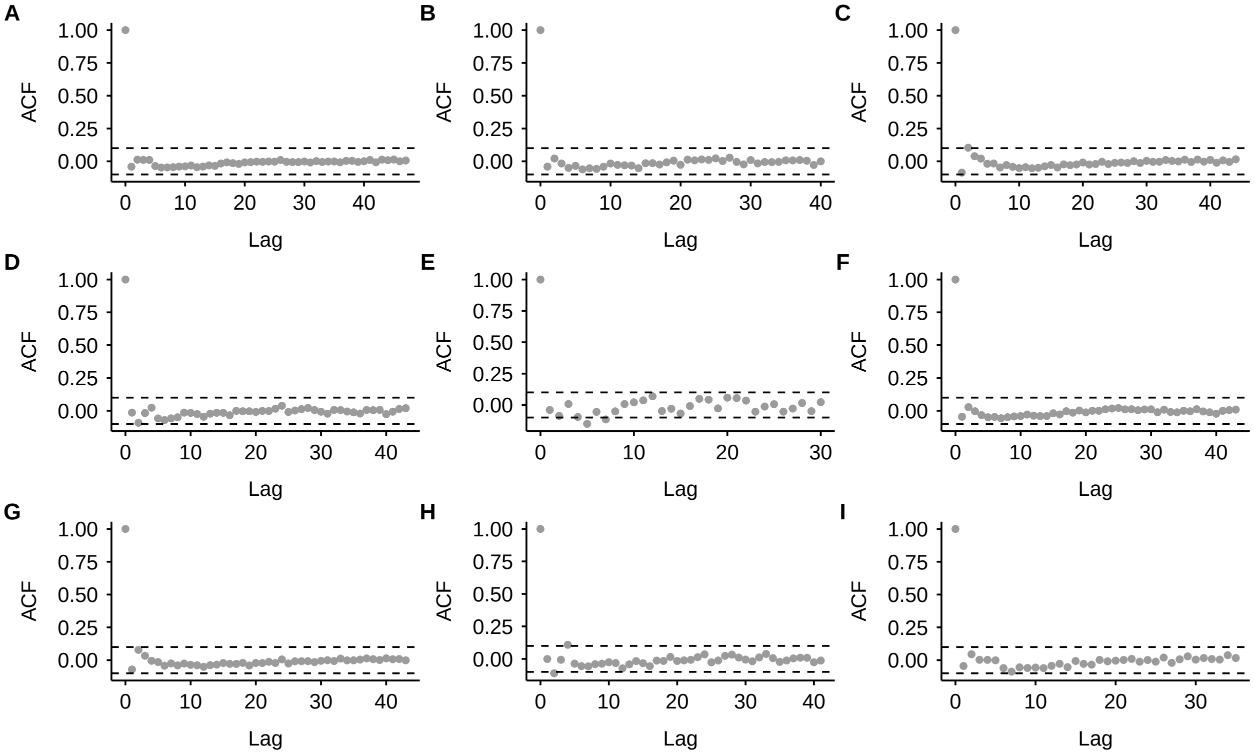

Figure S17: Autocorrelation of the standardised residuals for the multilevel Bayesian models. Limited evidence for autocorrelation is present for the (A) global model, (B-D) system specific models (freshwater, marine, terrestrial), and (E-I) amphibians, birds, fishes, mammals, and reptiles respectively.

Table S1: Model coefficients for global population trends. Median represents the median of the posterior distribution. CI low and high are the lower and higher values of the 95% credible interval.** Rhat is the ratio of the effective sample size to the overall number of iterations, with values close to one indicating convergence values.

Parameter	Median	CI_low	CI_high	Rhat	ESS
Intercept	-0.002	-0.011	0.008	1	16766.77
scaled_year	0.015	-0.154	0.180	1	5239.48
pollution1	0.002	-0.076	0.078	1	6679.82
habitatl1	-0.002	-0.025	0.021	1	11880.81
climatechange1	-0.006	-0.053	0.043	1	10845.48
invasive1	0.001	-0.052	0.054	1	9582.01
exploitation1	-0.002	-0.018	0.015	1	15430.68
disease1	0.003	-0.078	0.081	1	8577.92
pollution.habitatl1	0.002	-0.089	0.093	1	6692.33
pollution.climatechange1	0.011	-1.012	1.030	1	5600.75
pollution.invasive1	0.023	-1.019	1.055	1	5762.86
pollution.exploitation1	-0.018	-0.112	0.078	1	6942.95
pollution.disease1	-0.014	-0.263	0.239	1	8122.48
habitatl.climatechange1	0.007	-0.067	0.076	1	9522.42
habitatl.invasive1	0.002	-0.076	0.078	1	8657.07
habitatl.exploitation1	0.001	-0.039	0.042	1	11701.48
habitatl.disease1	0.008	-0.112	0.129	1	8171.24
climatechange.invasive1	-0.002	-0.295	0.299	1	8996.20
climatechange.exploitation1	0.010	-0.109	0.130	1	10428.10
climatechange.disease1	-0.009	-0.295	0.275	1	11005.17
invasive.exploitation1	-0.005	-0.101	0.096	1	9620.00
invasive.disease1	-0.037	-0.226	0.153	1	8536.11
exploitation.disease1	0.001	-0.192	0.196	1	8136.06
pollution.habitatl.climatechange1	-0.010	-1.043	1.019	1	5592.53
pollution.habitatl.invasive1	-0.023	-1.062	1.030	1	5684.72
pollution.habitatl.exploitation1	0.014	-0.110	0.141	1	6989.64
pollution.habitatl.disease1	-0.005	-0.288	0.291	1	7668.28
pollution.climatechange.invasive1	-0.025	-1.299	1.246	1	6833.08
pollution.climatechange.disease1	0.039	-1.000	1.112	1	5780.83
pollution.invasive.exploitation1	0.027	-1.003	1.082	1	5794.27
pollution.exploitation.disease1	0.035	-0.313	0.382	1	7420.34
habitatl.climatechange.invasive1	0.015	-0.328	0.356	1	8993.30
habitatl.climatechange.exploitation1	-0.019	-0.169	0.133	1	8989.49
habitatl.climatechange.disease1	0.002	-0.338	0.339	1	10009.82
habitatl.invasive.exploitation1	0.002	-0.161	0.162	1	10466.64
habitatl.invasive.disease1	0.030	-0.266	0.326	1	9265.35
habitatl.exploitation.disease1	-0.013	-0.273	0.250	1	8028.88
climatechange.invasive.exploitation1	0.006	-0.435	0.446	1	9521.38
invasive.exploitation.disease1	0.013	-0.359	0.388	1	9400.91
scaled_year:pollution1	-0.034	-0.068	0.001	1	3295.92
scaled_year:habitatl1	-0.021	-0.032	-0.011	1	5386.22
scaled_year:climatechange1	-0.047	-0.068	-0.025	1	4718.61
scaled_year:invasive1	-0.075	-0.101	-0.049	1	5220.89
scaled_year:exploitation1	-0.026	-0.035	-0.017	1	5244.63
scaled_year:disease1	-0.062	-0.097	-0.028	1	4449.70
scaled_year:pollution.habitatl1	0.044	0.004	0.085	1	3453.45
scaled_year:pollution.climatechange1	-0.016	-1.039	1.002	1	5746.41
scaled_year:pollution.invasive1	0.013	-0.967	1.033	1	5182.89
scaled_year:pollution.exploitation1	0.040	-0.003	0.082	1	3344.74
scaled_year:pollution.disease1	0.011	-0.104	0.128	1	4235.49
scaled_year:habitatl.climatechange1	0.040	0.009	0.071	1	4264.91
scaled_year:habitatl.invasive1	0.074	0.034	0.114	1	4961.77
scaled_year:habitatl.exploitation1	0.009	-0.010	0.027	1	4138.91
scaled_year:habitatl.disease1	0.084	0.024	0.142	1	4779.72
scaled_year:climatechange.invasive1	0.090	-0.058	0.235	1	6518.40
scaled_year:climatechange.exploitation1	0.055	0.006	0.104	1	6193.38
scaled_year:climatechange.disease1	0.112	-0.014	0.235	1	7038.99

Table S1: Model coefficients for global population trends. Median represents the median of the posterior distribution. CI low and high are the lower and higher values of the 95% credible interval.** Rhat is the ratio of the effective sample size to the overall number of iterations, with values close to one indicating convergence values. *(continued)*

Parameter	Median	CI_low	CI_high	Rhat	ESS
scaled__year:invasive.exploitation1	0.121	0.075	0.167	1	5631.31
scaled__year:invasive.disease1	0.115	0.035	0.196	1	5166.76
scaled__year:exploitation.disease1	0.012	-0.071	0.096	1	4328.87
scaled__year:pollution.habitatl.climatechange1	-0.017	-1.038	1.008	1	5769.50
scaled__year:pollution.habitatl.invasive1	-0.041	-1.065	0.952	1	5204.00
scaled__year:pollution.habitatl.exploitation1	-0.070	-0.126	-0.015	1	3742.15
scaled__year:pollution.habitatl.disease1	-0.041	-0.173	0.092	1	4344.52
scaled__year:pollution.climatechange.invasive1	0.154	-1.097	1.393	1	6081.11
scaled__year:pollution.climatechange.disease1	-0.159	-1.167	0.869	1	5775.97
scaled__year:pollution.invasive.exploitation1	-0.130	-1.155	0.860	1	5181.62
scaled__year:pollution.exploitation.disease1	0.083	-0.077	0.235	1	4300.89
scaled__year:habitatl.climatechange.invasive1	-0.070	-0.229	0.099	1	6275.61
scaled__year:habitatl.climatechange.exploitation1	-0.031	-0.097	0.037	1	5321.56
scaled__year:habitatl.climatechange.disease1	-0.166	-0.317	-0.018	1	6375.00
scaled__year:habitatl.invasive.exploitation1	-0.133	-0.207	-0.059	1	5567.15
scaled__year:habitatl.invasive.disease1	-0.152	-0.278	-0.027	1	5514.69
scaled__year:habitatl.exploitation.disease1	0.005	-0.106	0.116	1	4259.27
scaled__year:climatechange.invasive.exploitation1	-0.109	-0.322	0.103	1	7490.15
scaled__year:invasive.exploitation.disease1	-0.210	-0.365	-0.057	1	5027.81

Table S2: Model coefficients for global population trends across systems. Median represents the median of the posterior distribution. CI low and high are the lower and higher values of the 95% credible interval. Rhat is the ratio of the effective sample size to the overall number of iterations, with values close to one indicating convergence values.

System	Parameter	Median	CI_low	CI_high	Rhat	ESS
Freshwater	Intercept	-0.004	-0.030	0.022	1	12754.95
Freshwater	scaled_year	0.006	-0.116	0.134	1	5521.06
Freshwater	pollution1	-0.010	-0.143	0.124	1	7146.66
Freshwater	habitatl1	0.001	-0.045	0.048	1	12127.95
Freshwater	climatechange1	-0.010	-0.174	0.153	1	10972.94
Freshwater	invasive1	0.009	-0.091	0.103	1	8280.23
Freshwater	exploitation1	-0.001	-0.060	0.060	1	11073.57
Freshwater	disease1	-0.012	-0.209	0.180	1	12865.39
Freshwater	pollution.habitatl1	0.013	-0.136	0.163	1	7589.37
Freshwater	pollution.climatechange1	0.014	-0.391	0.426	1	13203.35
Freshwater	pollution.invasive1	-0.002	-0.373	0.358	1	10973.76
Freshwater	pollution.exploitation1	-0.005	-0.182	0.167	1	7431.10
Freshwater	pollution.disease1	0.023	-0.297	0.342	1	9581.91
Freshwater	habitatl.climatechange1	-0.001	-0.232	0.228	1	10561.24
Freshwater	habitatl.invasive1	0.001	-0.119	0.123	1	7862.94
Freshwater	habitatl.exploitation1	0.003	-0.090	0.099	1	10051.42
Freshwater	habitatl.disease1	0.008	-0.267	0.284	1	10721.85
Freshwater	climatechange.invasive1	-0.003	-0.393	0.380	1	11628.41
Freshwater	climatechange.exploitation1	0.018	-0.302	0.342	1	12398.20
Freshwater	climatechange.disease1	0.013	-0.267	0.297	1	11557.64
Freshwater	invasive.exploitation1	-0.009	-0.145	0.127	1	8448.46
Freshwater	exploitation.disease1	0.016	-0.327	0.365	1	10966.37
Freshwater	pollution.habitatl.invasive1	-0.004	-0.367	0.373	1	11072.99
Freshwater	pollution.habitatl.exploitation1	0.003	-0.202	0.215	1	7960.96
Freshwater	pollution.habitatl.disease1	-0.016	-0.377	0.342	1	10667.78
Freshwater	pollution.climatechange.disease1	0.011	-0.407	0.419	1	11130.40
Freshwater	pollution.exploitation.disease1	0.009	-0.390	0.404	1	9905.53
Freshwater	habitatl.climatechange.invasive1	-0.010	-0.396	0.366	1	11761.48
Freshwater	habitatl.climatechange.exploitation1	0.003	-0.349	0.345	1	11612.46
Freshwater	habitatl.climatechange.disease1	-0.002	-0.336	0.344	1	10364.05
Freshwater	habitatl.invasive.exploitation1	0.000	-0.239	0.239	1	10506.96
Freshwater	habitatl.exploitation.disease1	-0.007	-0.399	0.377	1	12460.22
Freshwater	scaled_year:pollution1	-0.069	-0.144	0.007	1	3654.67
Freshwater	scaled_year:habitatl1	-0.017	-0.042	0.007	1	2631.19
Freshwater	scaled_year:climatechange1	-0.056	-0.138	0.026	1	5280.14
Freshwater	scaled_year:invasive1	-0.089	-0.137	-0.043	1	3431.43
Freshwater	scaled_year:exploitation1	-0.003	-0.035	0.028	1	3903.79
Freshwater	scaled_year:disease1	-0.083	-0.184	0.015	1	5968.31
Freshwater	scaled_year:pollution.habitatl1	0.078	-0.009	0.161	1	3466.55
Freshwater	scaled_year:pollution.climatechange1	-0.044	-0.426	0.353	1	9896.34
Freshwater	scaled_year:pollution.invasive1	-0.025	-0.373	0.326	1	10254.28
Freshwater	scaled_year:pollution.exploitation1	0.015	-0.080	0.112	1	3966.57
Freshwater	scaled_year:pollution.disease1	0.013	-0.272	0.301	1	7130.72
Freshwater	scaled_year:habitatl.climatechange1	0.014	-0.115	0.143	1	5577.90
Freshwater	scaled_year:habitatl.invasive1	0.087	0.023	0.153	1	3644.98
Freshwater	scaled_year:habitatl.exploitation1	-0.026	-0.073	0.022	1	3440.82
Freshwater	scaled_year:habitatl.disease1	0.121	-0.056	0.301	1	6356.47
Freshwater	scaled_year:climatechange.invasive1	0.034	-0.316	0.390	1	9025.21
Freshwater	scaled_year:climatechange.exploitation1	0.071	-0.126	0.269	1	7651.56
Freshwater	scaled_year:climatechange.disease1	0.090	-0.087	0.268	1	5993.53
Freshwater	scaled_year:invasive.exploitation1	0.127	0.057	0.198	1	3487.54
Freshwater	scaled_year:exploitation.disease1	0.014	-0.313	0.337	1	7553.37
Freshwater	scaled_year:pollution.habitatl.invasive1	-0.017	-0.371	0.338	1	10481.86
Freshwater	scaled_year:pollution.habitatl.exploitation1	-0.034	-0.151	0.086	1	3636.54
Freshwater	scaled_year:pollution.habitatl.disease1	-0.041	-0.361	0.267	1	6823.34
Freshwater	scaled_year:pollution.climatechange.disease1	-0.040	-0.429	0.345	1	10929.37
Freshwater	scaled_year:pollution.exploitation.disease1	0.086	-0.281	0.451	1	8700.69

Table S2: Model coefficients for global population trends across systems. Median represents the median of the posterior distribution. CI low and high are the lower and higher values of the 95% credible interval. Rhat is the ratio of the effective sample size to the overall number of iterations, with values close to one indicating convergence values. *(continued)*

System	Parameter	Median	CI_low	CI_high	Rhat	ESS
Freshwater	scaled__year:habitatl.climatechange.invasive1	0.040	-0.317	0.392	1	9106.81
Freshwater	scaled__year:habitatl.climatechange.exploitation1	0.008	-0.228	0.241	1	6843.99
Freshwater	scaled__year:habitatl.climatechange.disease1	-0.164	-0.408	0.080	1	6026.35
Freshwater	scaled__year:habitatl.invasive.exploitation1	-0.202	-0.326	-0.078	1	4507.62
Freshwater	scaled__year:habitatl.exploitation.disease1	-0.075	-0.416	0.267	1	9330.24
Marine	Intercept	0.001	-0.015	0.018	1	18797.44
Marine	scaled__year	0.015	-0.113	0.136	1	6179.38
Marine	pollution1	0.009	-0.104	0.126	1	7315.41
Marine	habitatl1	-0.020	-0.065	0.026	1	11427.51
Marine	climatechange1	-0.016	-0.081	0.049	1	12419.27
Marine	invasive1	-0.041	-0.168	0.086	1	15226.51
Marine	exploitation1	-0.007	-0.030	0.016	1	17323.53
Marine	disease1	-0.002	-0.171	0.170	1	10525.81
Marine	pollution.habitatl1	0.005	-0.128	0.140	1	6915.88
Marine	pollution.climatechange1	0.004	-0.342	0.335	1	10109.16
Marine	pollution.invasive1	0.004	-0.338	0.341	1	12127.98
Marine	pollution.exploitation1	-0.014	-0.149	0.116	1	7317.14
Marine	pollution.disease1	-0.014	-0.258	0.223	1	9785.11
Marine	habitatl.climatechange1	0.031	-0.072	0.136	1	10426.30
Marine	habitatl.invasive1	0.027	-0.165	0.219	1	14157.95
Marine	habitatl.exploitation1	0.014	-0.058	0.086	1	10208.42
Marine	habitatl.disease1	0.026	-0.166	0.214	1	9775.90
Marine	climatechange.invasive1	0.017	-0.288	0.328	1	12221.42
Marine	climatechange.exploitation1	0.019	-0.110	0.145	1	11887.43
Marine	invasive.exploitation1	0.042	-0.169	0.249	1	10915.28
Marine	invasive.disease1	0.008	-0.336	0.352	1	12886.71
Marine	exploitation.disease1	0.007	-0.250	0.269	1	11304.97
Marine	pollution.habitatl.climatechange1	-0.014	-0.364	0.338	1	10969.60
Marine	pollution.habitatl.exploitation1	-0.001	-0.178	0.173	1	7490.56
Marine	pollution.habitatl.disease1	-0.016	-0.308	0.272	1	10275.71
Marine	pollution.climatechange.invasive1	0.008	-0.402	0.415	1	13669.66
Marine	pollution.invasive.exploitation1	-0.013	-0.387	0.354	1	12165.17
Marine	pollution.exploitation.disease1	0.006	-0.314	0.330	1	11351.94
Marine	habitatl.climatechange.invasive1	0.007	-0.375	0.398	1	16826.52
Marine	habitatl.climatechange.exploitation1	-0.048	-0.226	0.132	1	10347.15
Marine	habitatl.invasive.exploitation1	-0.003	-0.277	0.271	1	10802.03
Marine	habitatl.invasive.disease1	0.008	-0.373	0.389	1	14973.33
Marine	habitatl.exploitation.disease1	-0.012	-0.384	0.360	1	16424.58
Marine	climatechange.invasive.exploitation1	-0.009	-0.375	0.352	1	13443.44
Marine	invasive.exploitation.disease1	0.001	-0.386	0.380	1	13258.15
Marine	scaled__year:pollution1	-0.038	-0.091	0.014	1	3927.99
Marine	scaled__year:habitatl1	-0.020	-0.040	-0.001	1	4770.87
Marine	scaled__year:climatechange1	-0.028	-0.055	0.000	1	4786.87
Marine	scaled__year:invasive1	-0.068	-0.119	-0.015	1	6076.94
Marine	scaled__year:exploitation1	-0.032	-0.043	-0.021	1	4280.51
Marine	scaled__year:disease1	-0.048	-0.117	0.021	1	5552.37
Marine	scaled__year:pollution.habitatl1	0.064	-0.001	0.127	1	3780.68
Marine	scaled__year:pollution.climatechange1	0.024	-0.288	0.342	1	9459.37
Marine	scaled__year:pollution.invasive1	0.041	-0.285	0.361	1	9648.83
Marine	scaled__year:pollution.exploitation1	0.072	0.012	0.130	1	3646.36
Marine	scaled__year:pollution.disease1	0.101	-0.032	0.233	1	5730.75
Marine	scaled__year:habitatl.climatechange1	0.030	-0.014	0.073	1	4042.20
Marine	scaled__year:habitatl.invasive1	0.063	-0.023	0.149	1	6358.84
Marine	scaled__year:habitatl.exploitation1	0.034	0.004	0.064	1	4774.11
Marine	scaled__year:habitatl.disease1	0.060	-0.030	0.151	1	5683.81
Marine	scaled__year:climatechange.invasive1	0.039	-0.242	0.312	1	7410.19
Marine	scaled__year:climatechange.exploitation1	0.041	-0.013	0.095	1	5709.23
Marine	scaled__year:invasive.exploitation1	0.091	-0.013	0.195	1	7375.35

Table S2: Model coefficients for global population trends across systems. Median represents the median of the posterior distribution. CI low and high are the lower and higher values of the 95% credible interval. Rhat is the ratio of the effective sample size to the overall number of iterations, with values close to one indicating convergence values. *(continued)*

System	Parameter	Median	CI_low	CI_high	Rhat	ESS
Marine	scaled_year:invasive.disease1	0.020	-0.280	0.320	1	8839.66
Marine	scaled_year:exploitation.disease1	0.022	-0.100	0.146	1	6834.71
Marine	scaled_year:pollution.habitatl.climatechange1	-0.086	-0.407	0.232	1	9502.83
Marine	scaled_year:pollution.habitatl.exploitation1	-0.152	-0.230	-0.072	1	3743.66
Marine	scaled_year:pollution.habitatl.disease1	-0.115	-0.286	0.052	1	5532.57
Marine	scaled_year:pollution.climatechange.invasive1	0.104	-0.276	0.494	1	11137.36
Marine	scaled_year:pollution.invasive.exploitation1	-0.080	-0.406	0.248	1	9522.38
Marine	scaled_year:pollution.exploitation.disease1	-0.032	-0.214	0.146	1	6262.19
Marine	scaled_year:habitatl.climatechange.invasive1	-0.041	-0.339	0.261	1	8158.14
Marine	scaled_year:habitatl.climatechange.exploitation1	-0.054	-0.134	0.028	1	4861.83
Marine	scaled_year:habitatl.invasive.exploitation1	-0.031	-0.178	0.122	1	6852.32
Marine	scaled_year:habitatl.invasive.disease1	0.002	-0.308	0.320	1	9101.91
Marine	scaled_year:habitatl.exploitation.disease1	-0.011	-0.205	0.184	1	8130.89
Marine	scaled_year:climatechange.invasive.exploitation1	-0.035	-0.328	0.255	1	7910.46
Marine	scaled_year:invasive.exploitation.disease1	0.006	-0.305	0.318	1	9352.43
Terrestrial	Intercept	-0.002	-0.014	0.011	1	14493.64
Terrestrial	scaled_year	0.027	-0.168	0.223	1	4434.99
Terrestrial	pollution1	0.005	-0.101	0.109	1	9374.26
Terrestrial	habitatl1	0.002	-0.029	0.032	1	13425.78
Terrestrial	climatechange1	0.001	-0.070	0.071	1	12328.32
Terrestrial	invasive1	0.003	-0.063	0.071	1	12510.32
Terrestrial	exploitation1	0.000	-0.046	0.047	1	11955.00
Terrestrial	disease1	0.005	-0.077	0.087	1	11480.46
Terrestrial	pollution.habitatl1	-0.003	-0.139	0.134	1	9142.36
Terrestrial	pollution.climatechange1	-0.002	-0.382	0.377	1	11445.65
Terrestrial	pollution.invasive1	0.036	-0.292	0.369	1	9421.51
Terrestrial	pollution.exploitation1	-0.022	-0.161	0.118	1	9204.04
Terrestrial	pollution.disease1	-0.026	-0.338	0.289	1	12020.10
Terrestrial	habitatl.climatechange1	-0.003	-0.095	0.092	1	11206.66
Terrestrial	habitatl.invasive1	-0.011	-0.128	0.107	1	11554.16
Terrestrial	habitatl.exploitation1	-0.002	-0.070	0.063	1	10422.58
Terrestrial	habitatl.disease1	0.008	-0.162	0.175	1	11278.90
Terrestrial	climatechange.invasive1	-0.001	-0.227	0.227	1	12684.44
Terrestrial	climatechange.exploitation1	0.003	-0.236	0.248	1	11323.32
Terrestrial	climatechange.disease1	-0.002	-0.369	0.382	1	12596.79
Terrestrial	invasive.exploitation1	-0.002	-0.218	0.213	1	13150.68
Terrestrial	invasive.disease1	-0.025	-0.189	0.138	1	12321.57
Terrestrial	exploitation.disease1	-0.008	-0.186	0.171	1	12451.48
Terrestrial	pollution.habitatl.climatechange1	0.000	-0.376	0.379	1	10559.86
Terrestrial	pollution.habitatl.invasive1	-0.022	-0.371	0.337	1	10092.42
Terrestrial	pollution.habitatl.exploitation1	0.022	-0.238	0.282	1	12526.66
Terrestrial	pollution.habitatl.disease1	0.000	-0.344	0.331	1	11984.15
Terrestrial	pollution.invasive.exploitation1	0.047	-0.345	0.432	1	12009.52
Terrestrial	habitatl.climatechange.invasive1	0.031	-0.251	0.310	1	11881.08
Terrestrial	habitatl.climatechange.exploitation1	-0.004	-0.272	0.252	1	10582.68
Terrestrial	habitatl.climatechange.disease1	-0.002	-0.386	0.363	1	11457.51
Terrestrial	habitatl.invasive.exploitation1	-0.008	-0.283	0.278	1	11930.29
Terrestrial	habitatl.invasive.disease1	0.018	-0.257	0.294	1	11676.28
Terrestrial	habitatl.exploitation.disease1	-0.010	-0.269	0.249	1	10326.91
Terrestrial	invasive.exploitation.disease1	-0.022	-0.411	0.368	1	17636.14
Terrestrial	scaled_year:pollution1	-0.024	-0.079	0.029	1	3268.79
Terrestrial	scaled_year:habitatl1	-0.028	-0.043	-0.014	1	5017.55
Terrestrial	scaled_year:climatechange1	-0.069	-0.103	-0.037	1	4880.76
Terrestrial	scaled_year:invasive1	-0.081	-0.119	-0.041	1	5367.83
Terrestrial	scaled_year:exploitation1	-0.002	-0.023	0.021	1	5385.58
Terrestrial	scaled_year:disease1	-0.043	-0.083	-0.002	1	4499.32
Terrestrial	scaled_year:pollution.habitatl1	0.011	-0.061	0.082	1	3443.67

Table S2: Model coefficients for global population trends across systems. Median represents the median of the posterior distribution. CI low and high are the lower and higher values of the 95% credible interval. Rhat is the ratio of the effective sample size to the overall number of iterations, with values close to one indicating convergence values. *(continued)*

System	Parameter	Median	CI_low	CI_high	Rhat	ESS
Terrestrial	scaled__year:pollution.climatechange1	-0.001	-0.361	0.354	1	10361.97
Terrestrial	scaled__year:pollution.invasive1	0.005	-0.288	0.300	1	7397.21
Terrestrial	scaled__year:pollution.exploitation1	-0.064	-0.140	0.014	1	3747.35
Terrestrial	scaled__year:pollution.disease1	-0.242	-0.408	-0.071	1	6403.90
Terrestrial	scaled__year:habitatl.climatechange1	0.061	0.015	0.107	1	4222.72
Terrestrial	scaled__year:habitatl.invasive1	0.058	-0.003	0.119	1	4876.14
Terrestrial	scaled__year:habitatl.exploitation1	-0.014	-0.046	0.016	1	4832.00
Terrestrial	scaled__year:habitatl.disease1	0.069	-0.016	0.154	1	5307.78
Terrestrial	scaled__year:climatechange.invasive1	0.090	-0.059	0.238	1	6733.50
Terrestrial	scaled__year:climatechange.exploitation1	0.023	-0.111	0.152	1	6621.00
Terrestrial	scaled__year:climatechange.disease1	-0.013	-0.368	0.343	1	11160.56
Terrestrial	scaled__year:invasive.exploitation1	-0.042	-0.156	0.071	1	7719.45
Terrestrial	scaled__year:invasive.disease1	0.078	-0.008	0.162	1	4533.76
Terrestrial	scaled__year:exploitation.disease1	-0.044	-0.140	0.051	1	5243.55
Terrestrial	scaled__year:pollution.habitatl.climatechange1	0.001	-0.352	0.356	1	10235.70
Terrestrial	scaled__year:pollution.habitatl.invasive1	0.027	-0.278	0.326	1	7482.68
Terrestrial	scaled__year:pollution.habitatl.exploitation1	0.127	-0.002	0.253	1	4940.92
Terrestrial	scaled__year:pollution.habitatl.disease1	0.204	0.007	0.393	1	6253.15
Terrestrial	scaled__year:pollution.invasive.exploitation1	-0.032	-0.356	0.292	1	7957.89
Terrestrial	scaled__year:habitatl.climatechange.invasive1	-0.069	-0.246	0.108	1	6652.07
Terrestrial	scaled__year:habitatl.climatechange.exploitation1	0.013	-0.134	0.163	1	5754.05
Terrestrial	scaled__year:habitatl.climatechange.disease1	-0.007	-0.360	0.350	1	11127.80
Terrestrial	scaled__year:habitatl.invasive.exploitation1	0.054	-0.093	0.205	1	7178.80
Terrestrial	scaled__year:habitatl.invasive.disease1	-0.132	-0.283	0.020	1	5421.63
Terrestrial	scaled__year:habitatl.exploitation.disease1	0.079	-0.056	0.211	1	5032.60
Terrestrial	scaled__year:invasive.exploitation.disease1	-0.263	-0.479	-0.046	1	7547.80

Table S3: Model coefficients for global population trends across taxa. Median represents the median of the posterior distribution. CI low and high are the lower and higher values of the 95% credible interval. Rhat is the ratio of the effective sample size to the overall number of iterations, with values close to one indicating convergence values.

Taxon	Parameter	Median	CI_low	CI_high	Rhat	ESS
Amphibian	Intercept	-0.001	-0.066	0.064	1	14579.36
Amphibian	scaled_year	0.007	-0.083	0.134	1	3694.44
Amphibian	pollution1	-0.005	-0.300	0.289	1	9882.46
Amphibian	habitatl1	-0.002	-0.137	0.136	1	13599.16
Amphibian	climatechange1	0.002	-0.410	0.425	1	12117.86
Amphibian	invasive1	-0.008	-0.172	0.156	1	14231.75
Amphibian	exploitation1	0.007	-0.401	0.410	1	13772.79
Amphibian	disease1	0.007	-0.304	0.325	1	8199.11
Amphibian	pollution.habitatl1	-0.018	-0.346	0.309	1	9290.12
Amphibian	pollution.climatechange1	0.003	-0.440	0.456	1	11816.81
Amphibian	pollution.exploitation1	0.012	-0.440	0.448	1	14004.20
Amphibian	pollution.disease1	0.003	-0.430	0.449	1	12466.40
Amphibian	habitatl.climatechange1	-0.005	-0.427	0.421	1	13770.74
Amphibian	habitatl.invasive1	0.008	-0.330	0.347	1	12400.51
Amphibian	habitatl.exploitation1	0.009	-0.440	0.454	1	12170.11
Amphibian	habitatl.disease1	0.007	-0.314	0.337	1	8708.66
Amphibian	climatechange.disease1	0.004	-0.418	0.414	1	13073.73
Amphibian	invasive.exploitation1	-0.012	-0.464	0.436	1	14937.05
Amphibian	invasive.disease1	-0.017	-0.356	0.321	1	10051.85
Amphibian	exploitation.disease1	-0.016	-0.467	0.447	1	15948.48
Amphibian	pollution.habitatl.exploitation1	0.012	-0.429	0.449	1	14701.47
Amphibian	pollution.climatechange.disease1	0.005	-0.449	0.466	1	13396.04
Amphibian	habitatl.climatechange.disease1	-0.008	-0.432	0.417	1	12256.46
Amphibian	habitatl.invasive.disease1	0.007	-0.400	0.403	1	13050.00
Amphibian	invasive.exploitation.disease1	-0.013	-0.460	0.434	1	15033.52
Amphibian	scaled_year:pollution1	-0.206	-0.418	0.000	1	6658.07
Amphibian	scaled_year:habitatl1	-0.004	-0.080	0.074	1	4933.38
Amphibian	scaled_year:climatechange1	-0.013	-0.422	0.393	1	12429.84
Amphibian	scaled_year:invasive1	-0.043	-0.159	0.063	1	4073.63
Amphibian	scaled_year:exploitation1	-0.031	-0.428	0.363	1	11943.78
Amphibian	scaled_year:disease1	0.011	-0.266	0.282	1	5081.27
Amphibian	scaled_year:pollution.habitatl1	0.027	-0.201	0.257	1	6207.57
Amphibian	scaled_year:pollution.climatechange1	-0.004	-0.452	0.425	1	11973.56
Amphibian	scaled_year:pollution.exploitation1	0.055	-0.372	0.486	1	12214.14
Amphibian	scaled_year:pollution.disease1	-0.006	-0.442	0.438	1	13487.54
Amphibian	scaled_year:habitatl.climatechange1	-0.016	-0.425	0.400	1	12998.10
Amphibian	scaled_year:habitatl.invasive1	0.007	-0.222	0.238	1	6750.96
Amphibian	scaled_year:habitatl.exploitation1	0.056	-0.362	0.487	1	13253.52
Amphibian	scaled_year:habitatl.disease1	-0.027	-0.311	0.249	1	5498.01
Amphibian	scaled_year:climatechange.disease1	-0.008	-0.423	0.408	1	11342.39
Amphibian	scaled_year:invasive.exploitation1	-0.091	-0.523	0.340	1	12842.61
Amphibian	scaled_year:invasive.disease1	-0.026	-0.298	0.250	1	5245.63
Amphibian	scaled_year:exploitation.disease1	-0.094	-0.519	0.339	1	12950.98
Amphibian	scaled_year:pollution.habitatl.exploitation1	0.054	-0.378	0.492	1	13537.20
Amphibian	scaled_year:pollution.climatechange.disease1	-0.006	-0.433	0.427	1	12123.34
Amphibian	scaled_year:habitatl.climatechange.disease1	-0.011	-0.424	0.406	1	10203.33
Amphibian	scaled_year:habitatl.invasive.disease1	-0.065	-0.398	0.278	1	7533.38
Amphibian	scaled_year:invasive.exploitation.disease1	-0.092	-0.523	0.349	1	13267.58
Bird	Intercept	-0.002	-0.016	0.011	1	16717.18
Bird	scaled_year	0.025	-0.164	0.209	1	10510.46
Bird	pollution1	-0.001	-0.081	0.078	1	12422.21
Bird	habitatl1	-0.002	-0.029	0.025	1	19065.69
Bird	climatechange1	0.000	-0.054	0.053	1	19004.58
Bird	invasive1	0.010	-0.063	0.082	1	17879.56
Bird	exploitation1	0.002	-0.055	0.055	1	13831.46
Bird	disease1	-0.005	-0.213	0.207	1	8187.61
Bird	pollution.habitatl1	0.006	-0.088	0.100	1	12197.52

Table S3: Model coefficients for global population trends across taxa. Median represents the median of the posterior distribution. CI low and high are the lower and higher values of the 95% credible interval. Rhat is the ratio of the effective sample size to the overall number of iterations, with values close to one indicating convergence values. *(continued)*

Taxon	Parameter	Median	CI_low	CI_high	Rhat	ESS
Bird	pollution.climatechange1	-0.003	-0.318	0.317	1	16348.85
Bird	pollution.invasive1	0.012	-0.264	0.293	1	14153.07
Bird	pollution.exploitation1	-0.013	-0.125	0.104	1	10420.23
Bird	pollution.disease1	-0.001	-0.239	0.235	1	9526.25
Bird	habitatl.climatechange1	0.001	-0.077	0.078	1	17127.92
Bird	habitatl.invasive1	-0.007	-0.113	0.098	1	16592.59
Bird	habitatl.exploitation1	0.003	-0.071	0.080	1	14004.14
Bird	habitatl.disease1	0.011	-0.195	0.224	1	8619.98
Bird	climatechange.invasive1	-0.009	-0.225	0.206	1	16500.64
Bird	climatechange.exploitation1	0.003	-0.149	0.158	1	17521.60
Bird	climatechange.disease1	-0.006	-0.258	0.247	1	11131.63
Bird	invasive.exploitation1	0.002	-0.183	0.190	1	16556.68
Bird	invasive.disease1	0.000	-0.317	0.316	1	15972.68
Bird	exploitation.disease1	0.000	-0.249	0.255	1	12401.71
Bird	pollution.habitatl.climatechange1	0.001	-0.333	0.324	1	15654.29
Bird	pollution.habitatl.invasive1	-0.008	-0.315	0.291	1	14886.59
Bird	pollution.habitatl.exploitation1	0.001	-0.149	0.150	1	10358.20
Bird	pollution.habitatl.disease1	-0.013	-0.269	0.240	1	10514.22
Bird	pollution.climatechange.invasive1	-0.008	-0.395	0.387	1	15438.46
Bird	pollution.invasive.exploitation1	0.015	-0.310	0.332	1	13564.28
Bird	pollution.exploitation.disease1	0.024	-0.280	0.331	1	14111.12
Bird	habitatl.climatechange.invasive1	0.012	-0.262	0.282	1	16145.15
Bird	habitatl.climatechange.exploitation1	-0.005	-0.215	0.208	1	17138.97
Bird	habitatl.climatechange.disease1	0.001	-0.285	0.290	1	13146.66
Bird	habitatl.invasive.exploitation1	0.000	-0.237	0.232	1	15914.43
Bird	habitatl.invasive.disease1	-0.005	-0.341	0.329	1	16488.54
Bird	habitatl.exploitation.disease1	-0.008	-0.311	0.291	1	14134.10
Bird	climatechange.invasive.exploitation1	-0.002	-0.336	0.321	1	18670.21
Bird	invasive.exploitation.disease1	-0.003	-0.382	0.366	1	16966.73
Bird	scaled__year:pollution1	-0.016	-0.061	0.030	1	5814.25
Bird	scaled__year:habitatl1	-0.022	-0.037	-0.007	1	6850.22
Bird	scaled__year:climatechange1	-0.056	-0.084	-0.027	1	6772.98
Bird	scaled__year:invasive1	-0.074	-0.129	-0.019	1	6534.58
Bird	scaled__year:exploitation1	-0.031	-0.058	-0.003	1	6741.07
Bird	scaled__year:disease1	-0.043	-0.225	0.139	1	5267.55
Bird	scaled__year:pollution.habitatl1	0.015	-0.040	0.069	1	5686.33
Bird	scaled__year:pollution.climatechange1	0.051	-0.261	0.350	1	14565.93
Bird	scaled__year:pollution.invasive1	0.003	-0.269	0.278	1	10709.03
Bird	scaled__year:pollution.exploitation1	-0.006	-0.073	0.063	1	5918.79
Bird	scaled__year:pollution.disease1	-0.015	-0.213	0.172	1	5584.58
Bird	scaled__year:habitatl.climatechange1	0.029	-0.010	0.069	1	6413.97
Bird	scaled__year:habitatl.invasive1	0.096	0.029	0.163	1	6866.55
Bird	scaled__year:habitatl.exploitation1	-0.005	-0.043	0.033	1	6490.84
Bird	scaled__year:habitatl.disease1	0.047	-0.133	0.235	1	5401.86
Bird	scaled__year:climatechange.invasive1	0.072	-0.073	0.216	1	10272.22
Bird	scaled__year:climatechange.exploitation1	0.043	-0.040	0.125	1	8899.76
Bird	scaled__year:climatechange.disease1	0.091	-0.114	0.292	1	6247.30
Bird	scaled__year:invasive.exploitation1	0.068	-0.041	0.180	1	9095.51
Bird	scaled__year:invasive.disease1	0.016	-0.281	0.316	1	13995.67
Bird	scaled__year:exploitation.disease1	-0.012	-0.205	0.185	1	5865.03
Bird	scaled__year:pollution.habitatl.climatechange1	-0.053	-0.362	0.263	1	14646.26
Bird	scaled__year:pollution.habitatl.invasive1	-0.069	-0.351	0.210	1	10682.42
Bird	scaled__year:pollution.habitatl.exploitation1	0.015	-0.069	0.100	1	6068.10
Bird	scaled__year:pollution.habitatl.disease1	0.012	-0.192	0.211	1	5968.07
Bird	scaled__year:pollution.climatechange.invasive1	0.098	-0.267	0.453	1	14805.78
Bird	scaled__year:pollution.invasive.exploitation1	-0.033	-0.322	0.256	1	11241.73
Bird	scaled__year:pollution.exploitation.disease1	0.145	-0.077	0.370	1	7067.80
Bird	scaled__year:habitatl.climatechange.invasive1	-0.065	-0.236	0.108	1	10899.09

Table S3: Model coefficients for global population trends across taxa. Median represents the median of the posterior distribution. CI low and high are the lower and higher values of the 95% credible interval. Rhat is the ratio of the effective sample size to the overall number of iterations, with values close to one indicating convergence values. *(continued)*

Taxon	Parameter	Median	CI_low	CI_high	Rhat	ESS
Bird	scaled_year:habitatl.climatechange.exploitation1	0.033	-0.083	0.154	1	9777.83
Bird	scaled_year:habitatl.climatechange.disease1	-0.060	-0.275	0.159	1	7040.76
Bird	scaled_year:habitatl.invasive.exploitation1	-0.009	-0.149	0.128	1	8652.08
Bird	scaled_year:habitatl.invasive.disease1	-0.044	-0.349	0.263	1	13905.08
Bird	scaled_year:habitatl.exploitation.disease1	0.041	-0.173	0.260	1	6775.46
Bird	scaled_year:climatechange.invasive.exploitation1	-0.015	-0.234	0.207	1	11913.75
Bird	scaled_year:invasive.exploitation.disease1	0.052	-0.260	0.369	1	14335.59
Fish	Intercept	0.006	-0.018	0.030	1	16683.51
Fish	scaled_year	0.015	-0.047	0.065	1	3715.55
Fish	pollution1	-0.014	-0.229	0.206	1	6291.72
Fish	habitatl1	-0.046	-0.163	0.069	1	7616.80
Fish	climatechange1	-0.086	-0.252	0.088	1	12008.58
Fish	invasive1	-0.025	-0.144	0.091	1	8158.37
Fish	exploitation1	-0.012	-0.042	0.018	1	16879.51
Fish	disease1	-0.051	-0.275	0.172	1	17495.29
Fish	pollution.habitatl1	0.052	-0.188	0.286	1	6188.59
Fish	pollution.exploitation1	-0.067	-0.311	0.167	1	6377.71
Fish	habitatl.climatechange1	0.015	-0.318	0.357	1	9189.12
Fish	habitatl.invasive1	0.069	-0.099	0.239	1	6926.90
Fish	habitatl.exploitation1	0.036	-0.093	0.165	1	7599.65
Fish	climatechange.invasive1	0.008	-0.388	0.408	1	11575.30
Fish	climatechange.exploitation1	0.060	-0.199	0.319	1	12378.64
Fish	invasive.exploitation1	0.018	-0.135	0.168	1	8219.79
Fish	pollution.habitatl.exploitation1	0.036	-0.252	0.329	1	7468.88
Fish	habitatl.climatechange.invasive1	0.008	-0.395	0.405	1	12068.20
Fish	habitatl.climatechange.exploitation1	-0.005	-0.360	0.340	1	8969.69
Fish	habitatl.invasive.exploitation1	-0.041	-0.295	0.221	1	9326.49
Fish	scaled_year:pollution1	-0.126	-0.251	-0.001	1	4771.62
Fish	scaled_year:habitatl1	-0.049	-0.095	-0.004	1	4269.67
Fish	scaled_year:climatechange1	0.023	-0.048	0.096	1	7835.41
Fish	scaled_year:invasive1	-0.057	-0.104	-0.009	1	4430.12
Fish	scaled_year:exploitation1	-0.029	-0.041	-0.018	1	3048.03
Fish	scaled_year:disease1	-0.108	-0.201	-0.016	1	7999.89
Fish	scaled_year:pollution.habitatl1	0.203	0.062	0.340	1	4508.85
Fish	scaled_year:pollution.exploitation1	0.054	-0.086	0.200	1	4458.06
Fish	scaled_year:habitatl.climatechange1	-0.004	-0.324	0.318	1	8167.18
Fish	scaled_year:habitatl.invasive1	0.014	-0.063	0.093	1	3860.06
Fish	scaled_year:habitatl.exploitation1	0.034	-0.015	0.084	1	4118.54
Fish	scaled_year:climatechange.invasive1	0.037	-0.359	0.422	1	10804.90
Fish	scaled_year:climatechange.exploitation1	0.014	-0.103	0.132	1	6748.65
Fish	scaled_year:invasive.exploitation1	0.121	0.055	0.188	1	4282.73
Fish	scaled_year:pollution.habitatl.exploitation1	-0.184	-0.343	-0.023	1	4507.96
Fish	scaled_year:habitatl.climatechange.invasive1	0.036	-0.354	0.429	1	11669.59
Fish	scaled_year:habitatl.climatechange.exploitation1	-0.039	-0.369	0.286	1	8267.37
Fish	scaled_year:habitatl.invasive.exploitation1	-0.163	-0.280	-0.054	1	4456.38
Mammal	Intercept	-0.003	-0.021	0.016	1	13849.69
Mammal	scaled_year	0.014	-0.136	0.163	1	6335.64
Mammal	pollution1	0.008	-0.134	0.154	1	12538.67
Mammal	habitatl1	0.005	-0.046	0.055	1	12550.43
Mammal	climatechange1	0.004	-0.113	0.122	1	13397.77
Mammal	invasive1	0.006	-0.109	0.121	1	13585.03
Mammal	exploitation1	-0.001	-0.056	0.055	1	12603.97
Mammal	disease1	0.006	-0.079	0.092	1	15148.06
Mammal	pollution.habitatl1	-0.001	-0.276	0.272	1	12096.11
Mammal	pollution.invasive1	-0.006	-0.423	0.403	1	15459.25
Mammal	pollution.exploitation1	-0.008	-0.216	0.195	1	12696.07
Mammal	pollution.disease1	-0.005	-0.337	0.330	1	11905.51

Table S3: Model coefficients for global population trends across taxa. Median represents the median of the posterior distribution. CI low and high are the lower and higher values of the 95% credible interval. Rhat is the ratio of the effective sample size to the overall number of iterations, with values close to one indicating convergence values. *(continued)*

Taxon	Parameter	Median	CI_low	CI_high	Rhat	ESS
Mammal	habitatl.climatechange1	0.006	-0.163	0.172	1	12451.62
Mammal	habitatl.invasive1	-0.012	-0.219	0.202	1	14760.28
Mammal	habitatl.exploitation1	-0.001	-0.098	0.097	1	11439.19
Mammal	habitatl.disease1	-0.008	-0.244	0.226	1	14406.44
Mammal	climatechange.invasive1	0.043	-0.374	0.455	1	15330.09
Mammal	climatechange.exploitation1	-0.005	-0.236	0.229	1	12960.58
Mammal	invasive.exploitation1	-0.006	-0.325	0.310	1	16644.23
Mammal	invasive.disease1	-0.029	-0.247	0.180	1	12164.63
Mammal	exploitation.disease1	0.001	-0.207	0.199	1	14913.80
Mammal	pollution.habitatl.invasive1	-0.005	-0.396	0.403	1	14763.66
Mammal	pollution.habitatl.exploitation1	0.004	-0.304	0.316	1	11095.23
Mammal	pollution.habitatl.disease1	-0.011	-0.399	0.379	1	12632.84
Mammal	pollution.exploitation.disease1	-0.009	-0.379	0.383	1	13231.85
Mammal	habitatl.climatechange.invasive1	0.038	-0.377	0.449	1	15400.49
Mammal	habitatl.climatechange.exploitation1	-0.011	-0.282	0.272	1	11722.41
Mammal	habitatl.invasive.exploitation1	-0.011	-0.359	0.338	1	14750.06
Mammal	habitatl.exploitation.disease1	-0.010	-0.316	0.294	1	11029.71
Mammal	scaled_year:pollution1	-0.013	-0.066	0.040	1	8083.43
Mammal	scaled_year:habitatl1	-0.019	-0.039	0.001	1	7068.25
Mammal	scaled_year:climatechange1	-0.054	-0.100	-0.009	1	6433.52
Mammal	scaled_year:invasive1	-0.011	-0.065	0.043	1	7280.79
Mammal	scaled_year:exploitation1	0.006	-0.016	0.028	1	7216.97
Mammal	scaled_year:disease1	-0.044	-0.077	-0.012	1	6331.93
Mammal	scaled_year:pollution.habitatl1	-0.001	-0.146	0.143	1	8285.16
Mammal	scaled_year:pollution.invasive1	0.043	-0.330	0.403	1	12613.31
Mammal	scaled_year:pollution.exploitation1	-0.005	-0.082	0.074	1	8791.91
Mammal	scaled_year:pollution.disease1	0.069	-0.227	0.364	1	8004.59
Mammal	scaled_year:habitatl.climatechange1	0.078	0.009	0.144	1	6868.76
Mammal	scaled_year:habitatl.invasive1	-0.027	-0.124	0.070	1	7884.33
Mammal	scaled_year:habitatl.exploitation1	-0.003	-0.041	0.035	1	5385.86
Mammal	scaled_year:habitatl.disease1	0.064	-0.032	0.160	1	8339.05
Mammal	scaled_year:climatechange.invasive1	-0.040	-0.406	0.317	1	11810.95
Mammal	scaled_year:climatechange.exploitation1	0.038	-0.060	0.138	1	6350.40
Mammal	scaled_year:invasive.exploitation1	-0.042	-0.216	0.134	1	10452.96
Mammal	scaled_year:invasive.disease1	0.050	-0.038	0.144	1	6312.30
Mammal	scaled_year:exploitation.disease1	-0.033	-0.121	0.058	1	7024.04
Mammal	scaled_year:pollution.habitatl.invasive1	0.044	-0.322	0.412	1	12659.47
Mammal	scaled_year:pollution.habitatl.exploitation1	-0.052	-0.216	0.111	1	8572.24
Mammal	scaled_year:pollution.habitatl.disease1	-0.040	-0.347	0.277	1	8442.23
Mammal	scaled_year:pollution.exploitation.disease1	0.109	-0.205	0.415	1	8176.10
Mammal	scaled_year:habitatl.climatechange.invasive1	-0.043	-0.407	0.317	1	11424.83
Mammal	scaled_year:habitatl.climatechange.exploitation1	-0.059	-0.184	0.066	1	5472.69
Mammal	scaled_year:habitatl.invasive.exploitation1	0.023	-0.179	0.230	1	9403.68
Mammal	scaled_year:habitatl.exploitation.disease1	0.046	-0.090	0.184	1	6512.25
Reptile	Intercept	-0.002	-0.045	0.043	1	9403.21
Reptile	scaled_year	0.022	-0.098	0.151	1	6275.05
Reptile	pollution1	-0.001	-0.258	0.247	1	6432.44
Reptile	habitatl1	0.003	-0.058	0.064	1	9122.16
Reptile	climatechange1	-0.002	-0.123	0.122	1	10525.23
Reptile	invasive1	0.000	-0.121	0.122	1	14371.84
Reptile	exploitation1	0.001	-0.057	0.057	1	10087.68
Reptile	disease1	-0.002	-0.430	0.428	1	23204.65
Reptile	pollution.habitatl1	0.004	-0.251	0.254	1	6535.76
Reptile	pollution.exploitation1	0.001	-0.249	0.255	1	6399.70
Reptile	pollution.disease1	-0.002	-0.440	0.447	1	19390.75
Reptile	habitatl.climatechange1	0.000	-0.153	0.152	1	10583.47
Reptile	habitatl.invasive1	-0.005	-0.296	0.288	1	17859.94
Reptile	habitatl.exploitation1	-0.004	-0.096	0.086	1	8462.92

Table S3: Model coefficients for global population trends across taxa. Median represents the median of the posterior distribution. CI low and high are the lower and higher values of the 95% credible interval. Rhat is the ratio of the effective sample size to the overall number of iterations, with values close to one indicating convergence values. *(continued)*

Taxon	Parameter	Median	CI_low	CI_high	Rhat	ESS
Reptile	climatechange.exploitation1	0.005	-0.186	0.196	1	11827.55
Reptile	invasive.exploitation1	-0.003	-0.172	0.170	1	13565.71
Reptile	exploitation.disease1	0.000	-0.439	0.426	1	19662.54
Reptile	pollution.habitatl.exploitation1	0.000	-0.263	0.271	1	6823.35
Reptile	pollution.exploitation.disease1	0.000	-0.441	0.445	1	20507.86
Reptile	habitatl.climatechange.exploitation1	-0.001	-0.256	0.254	1	12761.64
Reptile	habitatl.invasive.exploitation1	0.000	-0.350	0.350	1	16846.98
Reptile	scaled__year:pollution1	0.065	-0.194	0.321	1	6678.58
Reptile	scaled__year:habitatl1	-0.031	-0.090	0.029	1	4620.64
Reptile	scaled__year:climatechange1	-0.055	-0.147	0.039	1	6422.67
Reptile	scaled__year:invasive1	-0.127	-0.217	-0.036	1	4336.09
Reptile	scaled__year:exploitation1	-0.021	-0.065	0.024	1	5205.74
Reptile	scaled__year:disease1	-0.011	-0.437	0.424	1	21747.24
Reptile	scaled__year:pollution.habitatl1	-0.003	-0.262	0.252	1	7018.00
Reptile	scaled__year:pollution.exploitation1	-0.027	-0.286	0.232	1	6692.22
Reptile	scaled__year:pollution.disease1	-0.008	-0.441	0.412	1	20641.41
Reptile	scaled__year:habitatl.climatechange1	0.080	-0.043	0.206	1	5429.01
Reptile	scaled__year:habitatl.invasive1	0.099	-0.122	0.319	1	9673.54
Reptile	scaled__year:habitatl.exploitation1	0.062	-0.017	0.141	1	5023.91
Reptile	scaled__year:climatechange.exploitation1	0.006	-0.130	0.144	1	6752.94
Reptile	scaled__year:invasive.exploitation1	0.096	-0.030	0.220	1	7399.39
Reptile	scaled__year:exploitation.disease1	-0.010	-0.435	0.419	1	20215.68
Reptile	scaled__year:pollution.habitatl.exploitation1	-0.095	-0.359	0.167	1	7013.51
Reptile	scaled__year:pollution.exploitation.disease1	-0.013	-0.436	0.413	1	18974.09
Reptile	scaled__year:habitatl.climatechange.exploitation1	-0.022	-0.215	0.173	1	6982.58
Reptile	scaled__year:habitatl.invasive.exploitation1	-0.019	-0.299	0.266	1	10276.18

Table S4: Proportion of threat interaction types estimated by the global model. n represents the number of interaction types present. Frequency represents the proportion of that given interaction type for that threat and system.

Threat	Interaction type	n	Frequency
Climate change	Synergistic	0	0.000
Climate change	Antagonistic	14	0.151
Climate change	Additive	79	0.849
Disease	Synergistic	0	0.000
Disease	Antagonistic	18	0.194
Disease	Additive	75	0.806
Exploitation	Synergistic	0	0.000
Exploitation	Antagonistic	11	0.108
Exploitation	Additive	91	0.892
Habitat loss	Synergistic	0	0.000
Habitat loss	Antagonistic	17	0.142
Habitat loss	Additive	103	0.858
Invasive	Synergistic	0	0.000
Invasive	Antagonistic	22	0.216
Invasive	Additive	80	0.784
Pollution	Synergistic	0	0.000
Pollution	Antagonistic	15	0.147
Pollution	Additive	87	0.853

Table S5: Proportion of threat interaction types across taxa. n represents the number of interaction types present Frequency represents the proportion of that given interaction type for that threat and taxa.

Threat	Taxon	Interaction type	n	Frequency
Climate change	Amphibian	Synergistic	0	0.000
Climate change	Amphibian	Antagonistic	0	0.000
Climate change	Amphibian	Additive	36	1.000
Climate change	Bird	Synergistic	0	0.000
Climate change	Bird	Antagonistic	15	0.179
Climate change	Bird	Additive	69	0.821
Climate change	Fish	Synergistic	0	0.000
Climate change	Fish	Antagonistic	3	0.083
Climate change	Fish	Additive	33	0.917
Climate change	Mammal	Synergistic	0	0.000
Climate change	Mammal	Antagonistic	2	0.056
Climate change	Mammal	Additive	34	0.944
Climate change	Reptile	Synergistic	0	0.000
Climate change	Reptile	Antagonistic	5	0.238
Climate change	Reptile	Additive	16	0.762
Disease	Amphibian	Synergistic	3	0.045
Disease	Amphibian	Antagonistic	0	0.000
Disease	Amphibian	Additive	63	0.955
Disease	Bird	Synergistic	0	0.000
Disease	Bird	Antagonistic	9	0.107
Disease	Bird	Additive	75	0.893
Disease	Fish	Synergistic	NA	NA
Disease	Fish	Antagonistic	NA	NA
Disease	Fish	Additive	NA	NA
Disease	Mammal	Synergistic	0	0.000
Disease	Mammal	Antagonistic	9	0.176
Disease	Mammal	Additive	42	0.824
Disease	Reptile	Synergistic	0	0.000
Disease	Reptile	Antagonistic	0	0.000
Disease	Reptile	Additive	21	1.000
Exploitation	Amphibian	Synergistic	3	0.071
Exploitation	Amphibian	Antagonistic	0	0.000
Exploitation	Amphibian	Additive	39	0.929
Exploitation	Bird	Synergistic	0	0.000
Exploitation	Bird	Antagonistic	15	0.147
Exploitation	Bird	Additive	87	0.853
Exploitation	Fish	Synergistic	0	0.000
Exploitation	Fish	Antagonistic	14	0.275
Exploitation	Fish	Additive	37	0.725
Exploitation	Mammal	Synergistic	0	0.000
Exploitation	Mammal	Antagonistic	6	0.080
Exploitation	Mammal	Additive	69	0.920
Exploitation	Reptile	Synergistic	0	0.000
Exploitation	Reptile	Antagonistic	13	0.197
Exploitation	Reptile	Additive	53	0.803
Habitat loss	Amphibian	Synergistic	0	0.000
Habitat loss	Amphibian	Antagonistic	0	0.000
Habitat loss	Amphibian	Additive	57	1.000
Habitat loss	Bird	Synergistic	0	0.000
Habitat loss	Bird	Antagonistic	14	0.117
Habitat loss	Bird	Additive	106	0.883
Habitat loss	Fish	Synergistic	0	0.000
Habitat loss	Fish	Antagonistic	17	0.283
Habitat loss	Fish	Additive	43	0.717
Habitat loss	Mammal	Synergistic	0	0.000
Habitat loss	Mammal	Antagonistic	8	0.086
Habitat loss	Mammal	Additive	85	0.914
Habitat loss	Reptile	Synergistic	0	0.000
Habitat loss	Reptile	Antagonistic	13	0.255

Table S5: Proportion of threat interaction types across taxa. n represents the number of interaction types present Frequency represents the proportion of that given interaction type for that threat and taxa. *(continued)*

Threat	Taxon	Interaction type	n	Frequency
Habitat loss	Reptile	Additive	38	0.745
Invasive	Amphibian	Synergistic	3	0.083
Invasive	Amphibian	Antagonistic	0	0.000
Invasive	Amphibian	Additive	33	0.917
Invasive	Bird	Synergistic	0	0.000
Invasive	Bird	Antagonistic	23	0.225
Invasive	Bird	Additive	79	0.775
Invasive	Fish	Synergistic	0	0.000
Invasive	Fish	Antagonistic	9	0.250
Invasive	Fish	Additive	27	0.750
Invasive	Mammal	Synergistic	0	0.000
Invasive	Mammal	Antagonistic	0	0.000
Invasive	Mammal	Additive	57	1.000
Invasive	Reptile	Synergistic	0	0.000
Invasive	Reptile	Antagonistic	8	0.381
Invasive	Reptile	Additive	13	0.619
Pollution	Amphibian	Synergistic	0	0.000
Pollution	Amphibian	Antagonistic	0	0.000
Pollution	Amphibian	Additive	42	1.000
Pollution	Bird	Synergistic	0	0.000
Pollution	Bird	Antagonistic	9	0.097
Pollution	Bird	Additive	84	0.903
Pollution	Fish	Synergistic	0	0.000
Pollution	Fish	Antagonistic	12	0.571
Pollution	Fish	Additive	9	0.429
Pollution	Mammal	Synergistic	0	0.000
Pollution	Mammal	Antagonistic	6	0.100
Pollution	Mammal	Additive	54	0.900
Pollution	Reptile	Synergistic	0	0.000
Pollution	Reptile	Antagonistic	0	0.000
Pollution	Reptile	Additive	36	1.000

Table S6: Proportion of threat interaction types across systems. n represents the number of interaction types present. Frequency represents the proportion of that given interaction type for that threat and system.

Threat	System	Interaction type	n	Frequency
Climate change	Freshwater	Synergistic	0	0.000
Climate change	Freshwater	Antagonistic	8	0.121
Climate change	Freshwater	Additive	58	0.879
Climate change	Marine	Synergistic	0	0.000
Climate change	Marine	Antagonistic	12	0.174
Climate change	Marine	Additive	57	0.826
Climate change	Terrestrial	Synergistic	0	0.000
Climate change	Terrestrial	Antagonistic	6	0.091
Climate change	Terrestrial	Additive	60	0.909
Disease	Freshwater	Synergistic	0	0.000
Disease	Freshwater	Antagonistic	13	0.188
Disease	Freshwater	Additive	56	0.812
Disease	Marine	Synergistic	0	0.000
Disease	Marine	Antagonistic	20	0.290
Disease	Marine	Additive	49	0.710
Disease	Terrestrial	Synergistic	10	0.133
Disease	Terrestrial	Antagonistic	3	0.040
Disease	Terrestrial	Additive	62	0.827
Exploitation	Freshwater	Synergistic	0	0.000
Exploitation	Freshwater	Antagonistic	7	0.093
Exploitation	Freshwater	Additive	68	0.907
Exploitation	Marine	Synergistic	0	0.000
Exploitation	Marine	Antagonistic	28	0.275
Exploitation	Marine	Additive	74	0.725
Exploitation	Terrestrial	Synergistic	9	0.107
Exploitation	Terrestrial	Antagonistic	0	0.000
Exploitation	Terrestrial	Additive	75	0.893
Habitat loss	Freshwater	Synergistic	0	0.000
Habitat loss	Freshwater	Antagonistic	21	0.206
Habitat loss	Freshwater	Additive	81	0.794
Habitat loss	Marine	Synergistic	0	0.000
Habitat loss	Marine	Antagonistic	20	0.196
Habitat loss	Marine	Additive	82	0.804
Habitat loss	Terrestrial	Synergistic	0	0.000
Habitat loss	Terrestrial	Antagonistic	6	0.050
Habitat loss	Terrestrial	Additive	114	0.950
Invasive	Freshwater	Synergistic	0	0.000
Invasive	Freshwater	Antagonistic	15	0.294
Invasive	Freshwater	Additive	36	0.706
Invasive	Marine	Synergistic	0	0.000
Invasive	Marine	Antagonistic	29	0.312
Invasive	Marine	Additive	64	0.688
Invasive	Terrestrial	Synergistic	9	0.107
Invasive	Terrestrial	Antagonistic	3	0.036
Invasive	Terrestrial	Additive	72	0.857
Pollution	Freshwater	Synergistic	0	0.000
Pollution	Freshwater	Antagonistic	14	0.187
Pollution	Freshwater	Additive	61	0.813
Pollution	Marine	Synergistic	0	0.000
Pollution	Marine	Antagonistic	24	0.286
Pollution	Marine	Additive	60	0.714
Pollution	Terrestrial	Synergistic	7	0.093
Pollution	Terrestrial	Antagonistic	0	0.000
Pollution	Terrestrial	Additive	68	0.907
